# A human model of Buruli ulcer: Provisional protocol for a *Mycobacterium ulcerans *controlled human infection study.

**DOI:** 10.12688/wellcomeopenres.22719.1

**Published:** 2024-08-19

**Authors:** Stephen Muhi, Julia L. Marshall, Daniel P. O'Brien, Paul D.R. Johnson, Gayle Ross, Anand Ramakrishnan, Laura K. Mackay, Marcel Doerflinger, James S. McCarthy, Euzebiusz Jamrozik, Joshua Osowicki, Timothy P. Stinear

**Affiliations:** 1 Department of Microbiology and Immunology, Doherty Institute, University of Melbourne, Melbourne, Victoria, 3000, Australia; 2 Victorian Infectious Diseases Service, The Royal Melbourne Hospital, Parkville, Victoria, Australia; 3 Walter and Eliza Hall Institute of Medical Research, Melbourne, Victoria, Australia; 4 Department of General Medicine, The Royal Melbourne Hospital, Parkville, Victoria, Australia; 5 Department of Infectious Diseases, Doherty Institute, University of Melbourne, Melbourne, Victoria, Australia; 6 Department of Infectious Diseases, Barwon Health, Geelong, Victoria, Australia; 7 Austin Health, Heidelberg, Victoria, Australia; 8 Department of Dermatology, The Royal Melbourne Hospital, Parkville, Victoria, Australia; 9 Department of Plastic and Reconstructive Surgery, The Royal Melbourne Hospital, Parkville, Victoria, Australia; 10 Department of Medical Biology, University of Melbourne, Parkville, Victoria, Australia; 11 WHO Collaborating Centre for Bioethics, Monash University, Clayton, Victoria, Australia; 12 Tropical Diseases Research Group, Murdoch Children’s Research Institute, The Royal Children’s Hospital, Parkville, Victoria, Australia; 13 Infectious Diseases Unit, Department of General Medicine, The Royal Children's Hospital, Parkville, Victoria, Australia; 14 Department of Paediatrics, University of Melbourne, Parkville, Victoria, Australia; 15 WHO Collaborating Centre for Mycobacterium ulcerans, Doherty Institute, Melbourne, Victoria, Australia

**Keywords:** Buruli ulcer, Bairnsdale ulcer, Mycobacterium ulcerans, M. ulcerans, controlled human infection model

## Abstract

Critical knowledge gaps have impeded progress towards reducing the global burden of disease due to *Mycobacterium ulcerans*, the cause of the neglected tropical disease Buruli ulcer (BU). Development of a controlled human infection model of BU has been proposed as an experimental platform to explore host-pathogen interactions and evaluate tools for prevention, diagnosis, and treatment. We have previously introduced the use case for a new human model and identified *M. ulcerans* JKD8049 as a suitable challenge strain. Here, we present a provisional protocol for an initial study, for transparent peer review during the earliest stages of protocol development. Following simultaneous scientific peer review and community/stakeholder consultation of this provisional protocol, we aim to present a refined protocol for institutional review board (IRB) evaluation.

## Plain language summary

This paper describes a provisional clinical protocol for the pilot human challenge model of *Mycobacterium ulcerans* infection, which causes the skin disease 'Buruli ulcer' (BU). BU is typically painless and begins as a small area of redness or swelling, and is curable with antibiotics. If the diagnosis is delayed, it can result in large ulceration and disability. Side effects from antibiotics are common but rarely severe; nevertheless, preventative strategies, such as vaccination, are urgently needed. The overarching project, known as 'MuCHIM', aims to establish a safe and acceptable controlled human challenge model (CHIM) of this disease in healthy volunteers in Melbourne, Australia. This pilot protocol primarily aims to establish that it is safe and acceptable to participants, and secondarily to confirm successful establishment of infection and the infection rate amongst participants. We also aim to test less invasive diagnostic tests, assess immune responses to infection, to understand changes in the human microbiome during the trial, and explore microbiological characteristics of *M. ulcerans* infection. If this pilot is successful, we hope to test vaccines and other therapeutics using this model, which could blunt or reduce the rising incidence of this disease in Australia, while further informing vaccine development research.

## Background

*Mycobacterium ulcerans* is a slow-growing pathogen, typically causing indolent, painless, and progressive necrotising cutaneous ulcerative lesions known as ‘Buruli ulcer’ (BU), predominantly in Australia and West Africa. BU is classified as a neglected tropical disease by the World Health Organization ^1^, reflecting the unmet need for better strategies for treatment and prevention. Delayed diagnosis can lead to significant morbidity due to advanced ulceration, including contractures and deformity due to scarring (particularly over joints), and high costs to the healthcare system ^2^. In Australia, incidence continues to rise, and clusters have emerged in new locations beyond the borders of historically affected areas ^3, 4^. Antibiotic treatment is highly effective but prolonged, side effects are not uncommon ^5^, and reconstructive surgery may be required for severe lesions. Improved antibiotic regimens and preventative vaccines are important research priorities.

Although several vaccination targets have been identified, vaccine development has been impeded ^6^. Vaccination with *M. bovis* bacillus Calmette–Guérin (BCG) has shown at least short-term protection ^6^. Although the longevity of this response has been questioned in earlier trials in Africa ^6, 7^, recent Australian studies have shown significant protection from prior BCG vaccination ^7^, which has not been part of the routine Australian vaccination schedule since the mid-1980s. The relative geographic restriction and sporadic epidemiology of BU in Australia are major barriers to undertaking field trials to determine vaccine efficacy. For example, in an endemic setting such as the Bellarine Peninsula (in the state of Victoria) with an annual BU incidence of approximately 0.15%, a sample size of approximately 100,000 people would be required to detect a protective effect of BCG vaccination with 80% power (assuming ~40% vaccine efficacy) ^6, 8^. Given the long incubation period (4–5 months average) ^9, 10^ and slow clinical course of BU, a vaccine efficacy trial would likely be prohibitively lengthy and expensive.

A controlled human infection model (CHIM) of *Mycobacterium ulcerans* (‘MuCHIM’) in healthy adult volunteers would advance our understanding of human immune responses to *M. ulcerans* and could be an efficient platform for evaluating vaccines, chemoprophylaxis, and novel therapeutics. The following sample size calculation illustrates the potential of such a model: assuming 100% BU attack rate (with 80% power to detect a difference and p < 0.05 for statistical significance), a MuCHIM could detect a difference between two arms of just 14 participants for an investigational vaccine such as *M. bovis* BCG ^6^. This approach would overcome the research bottleneck limiting vaccine evaluation, and facilitate progression towards later stage clinical trials. A positive finding in a human model for an investigational vaccine ^6^ would support consideration of vaccine deployment to curb the rising incidence of BU in southeastern Australia. Likewise, if single-dose or short-course post-exposure antibiotic treatment was effective in preventing experimental human BU, then this may be an option for cohabitants of a BU case, due to the clustered nature of transmission ^11^.

Although *M. ulcerans* can infect a range of mammalian hosts, including experimental animals such as guinea pigs, its manifestations do not recapitulate key features of human BU ^12^. Immune responses to *M. ulcerans* have been studied most extensively in inbred laboratory mice. The clinical syndrome following *M. ulcerans* challenge varies across mice of different genetic backgrounds, with C57BL/6 mice exhibiting a more pronounced inflammatory response compared to BALB/c mice ^13^, while FVB/N mice recover spontaneously ^14^. Knowledge of immune responses to *M. ulcerans* in humans is limited by the retrospective and uncontrolled nature of studies, resulting in difficulties characterising immune correlates of protection and disease ^15^. A human model of BU disease would inform understanding of the immunopathology of BU, and potential pathways to immune protection or disease modification in the intended target human host.

CHIMs have been successfully and safely implemented for numerous infectious diseases ^16^. Several skin infection models also serve as opportunities for direct comparison, such as schistosomiasis ^17^, chancroid ^18^ and leishmaniasis ^19^. Compared to these infections, BU is typically localised to the skin and soft tissue and, to our knowledge, mortality has never been reported following natural infection of otherwise healthy young Australian adults.

In this proposed model, participants will be asked to adhere to a reference regimen of antibiotic therapy with rifampicin and clarithromycin, although treatment duration may be significantly abbreviated if surgical excision is performed. Even without surgery, the cure rate with antibiotic therapy is extremely high, with recurrence rarely reported ^20^. In participants who elect not to receive surgical excision, the trial is likely to leave a superficial scar not dissimilar to that following *M. bovis* BCG vaccination. Unlike clinical BU ^21^, participants will be subjected to rigorous follow up, ensuring very early diagnosis and immediate intervention. Hence, scarring or adverse outcomes are expected to be minimal compared to natural infection.

## Objectives and outcome measures

### Overarching aim

To establish a safe and acceptable controlled human infection model of BU in Melbourne, Victoria, Australia.

### Primary objective

Confirm safety, tolerability and cure of experimental *M. ulcerans* infection in healthy adult participants.

### Secondary objectives

1. Confirm *M. ulcerans* at the subcutaneous injection site by swab or biopsy
2. Establish a model with ≥ 60% infection rate

### Exploratory objectives

1. Determine safety and tolerability of a minimally-invasive biopsy of pre-ulcerative lesions, with an aim to employ less invasive methods for refined future protocols
2. Assess immune response to *M. ulcerans* locally in affected skin tissue and systemically in peripheral blood
3. Understand changes in aspects of the microbiome during and following antibiotic treatment (e.g., changes in organism populations and carriage of antimicrobial resistant bacteria)
4. Explore the microbiological features of *M. ulcerans* infection in healthy human volunteers

## Study design

MuCHIM involves a prospective longitudinal controlled human infection study, investigating subcutaneous injection of *M. ulcerans* JKD8049 in healthy adult volunteers. Development of MuCHIM will consist of two distinct stages. Stage 1 will include the establishment of policies and procedures required to optimise the safe and ethical conduct of the study. Stage 2A includes the first-in-human challenge of *M. ulcerans* (the ‘pilot’ challenge stage) and dose escalation, followed by dose confirmation in stage 2B.

### Stage 1

A. Community consultation (e.g., focus groups)
B. Establishment of an independent safety review committee
C. Creation of a working cell bank of *M. ulcerans* for human challenge
D. Quality control and release of challenge cell bank
E. Ethical and regulatory approval of the clinical trial

### Stage 2

A. Recruitment of three participants for first-in-human subcutaneous challenge with 10 – 20 colony forming units (CFU) of *M. ulcerans* JKD8049 in the medial forearm; dose escalation may be required to establish infection (see ‘Dose escalation’)
B. Recruitment of 10 participants for a dose confirmation study, using the lowest dose that successfully challenges ≥ 2 of 3 participants in Stage 2A

Parameters for progression (including dose escalation) across Stage 2A/B include:

▪ Participants able to tolerate study procedures
▪ No study-related serious adverse event
▪ Confirmation of intra-lesional *M. ulcerans* using IS *2404* PCR
▪ Successful resolution of infection in all participants (scarring may persist)

Parameters for progression to future vaccine/therapeutic trials:

▪ Above, plus establishment of WHO grade I lesion in ≥ 60% of Stage 2A/B participants

### Community consultation using focus groups

Australian individuals living in an endemic area, including those with previous BU, will be recruited for focus group discussion. A small group of clinicians with experience managing BU will also be invited to participate. Public engagement will allow a transparent dialogue and an opportunity to assess the acceptability of MuCHIM and understand its impact on the community. The aim of this qualitative research will be to understand the barriers and enablers to conducting a BU human infection model. The clinical trial protocol may therefore be informed by the learnings of this qualitative research. Focus group participants will also be invited to comment on draft participant information and consent forms, to ensure appropriate language and clarity is provided to potential participants.

### Sample size

The first-in-human trial will recruit 3 consecutive participants for preliminary safety and tolerability evaluation, followed by dose confirmation in 10 participants. Future applications of this model, designed to test interventions in randomised, double-blind trials, will be dependent on the attack rate estimated from Stage 2A and 2B of the study. Prior animal studies using a low dose (≤ 20 CFU) of the proposed challenge strain *M. ulcerans* JKD8049 have demonstrated an attack rate of 100% ^22^. It is unknown whether this will be the same in humans. Thus, the study design and power required for future applications will be analysed separately.

### Challenge site

Emulating the natural route of infection, the challenge procedure will involve a subcutaneous injection of the mycobacterial inoculum into the medial forearm approximately one-third of the distance from elbow flexor crease to the wrist, overlying the superficial flexor muscles ^23^. This site is favoured because:

1. It is a common site of natural infection ^24^
2. It is not associated with an increased risk of developing an oedematous lesion ^25^
3. There is minimal risk of (already very rare) contiguous spread to surrounding bone and joint structures ^26^
4. Contractures due to scarring would be highly unlikely
5. Surgical excision with primary wound closure will be possible
6. Any adverse aesthetic impact of scarring will be minimised
7. It is suitable for participants to examine and photograph themselves

**Figure 1.**
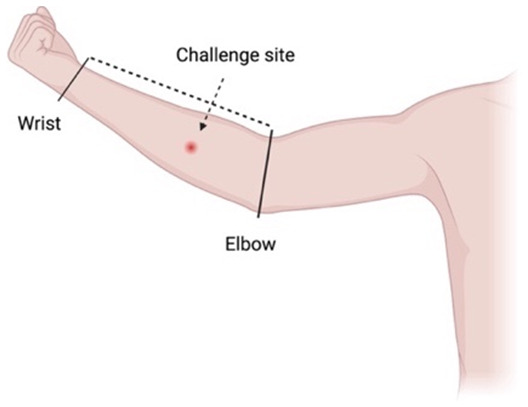
The proposed subcutaneous challenge site will be along the medial aspect of the forearm, one-third of the distance from the elbow to the wrist. Created with BioRender.com.

The challenge site will be defined by visible anatomical landmarks. The arm will be measured from the elbow crease to the wrist, and the challenge site will be estimated using this distance ( Figure 1). The non-dominant arm may be more amenable to self-inspection and dressing changes, although the side can be nominated by the participant.

## Recruitment and eligibility

### Participant recruitment

Following ethics approval, volunteers may be recruited using databases and/or advertising through posters, print, radio, or social media.

### Participant eligibility criteria

In Australia, most patients with BU are adults, therefore this study aims to recruit adults for study inclusion. In addition, BU disease severity and antibiotic complication rates are particularly problematic in children ^27^ and the elderly ^28^. The ethical considerations for recruiting children into CHIMs is complex ^29^, with a widespread presumption against enrolling children in such studies ^30^. Therefore, initial studies will include adults aged 18 to 45 years old (inclusive). As the duration of participation is lengthy, only participants that are foreseeably likely to comply for the study duration are eligible to participate. Measures to facilitate follow-up will be prioritised, such as the online capture of self-reported measures and digital photography of the challenge site using a portable electronic camera-enabled device.

### Inclusion criteria

●   Age between 18 and 45 years of age (inclusive) at the time of enrolment

●   Capacity to provide written informed consent

●   Willing and able to comply with all study requirements, including antibiotics

●   Planned residence near study site (≤ 2 hr drive or public transit) for at least 12 months from enrolment

●   English language proficiency (to ensure comprehensive understanding of the study and their proposed involvement)

●   Individuals of childbearing potential with a negative urine pregnancy test at screening and willing to practice acceptable contraception until 30 days after antibiotic completion ( Table 4)

●   Provides written consent to discuss medical history with, and to share correspondence with, their nominated general practitioner or other relevant health care provider

●   Up-to-date with tetanus vaccination, or willing to receive vaccination prior to challenge (as ulcers with devitalised tissue are considered tetanus-prone wounds ^31^)

### Temporary exclusion criteria

▪   Use of any antibiotic within 28 days of subcutaneous challenge

▪   Any vaccination within 28 days of subcutaneous challenge

▪   Febrile or other transient medical illness

### Exclusion criteria

▪   Clinically significant history of skin disorder, malignancy, cardiovascular disease, respiratory disease, gastrointestinal disease, liver disease, renal disease, endocrine disorder, haematological disease (including bleeding disorder) or neurological disease ^*^

▪   Clinically significant psychiatric disorder anticipated to interrupt follow-up ^*^

▪   Body mass index ≥ 25 kg/m ^2^

▪   Primary or secondary immunocompromise, based on history, examination and/or investigation

▪   Current or recent (within 3 months) habitual smoking, including cigarettes, cigars, e-cigarettes, vaping, or smoking of recreational drugs

▪   History of sustained harmful alcohol consumption, defined as ≥ 10 standard drinks per week within the past 12 months ^32^

▪   Unwilling or unable to abstain from alcohol consumption during antibiotic treatment

▪   Medication or other interaction with rifampicin, clarithromycin or fluroquinolones

▪   History of allergy to rifamycins, macrolides or fluroquinolones

▪   History of allergy to local anaesthetic

▪   History of allergy to corticosteroids

▪   Abnormal baseline electrocardiogram (ECG), and/or baseline QTc (Bazett) ≥ 430 ms (male) and ≥ 440 ms (female) measured in lead II of a standard 12-lead ECG ^33^

▪   History of hearing impairment or abnormal baseline audiometry

▪   For individuals of childbearing potential:

      ○   Current or planned pregnancy

      ○   Current or planned breastfeeding

      ○   Unwilling or unable to use acceptable contraception from time of challenge until 30 days after antibiotic completion ( Table 4)

▪   History of poor wound healing or excessive scarring ^34, 35^

▪   History of allergy to any challenge dose excipient

▪   Previous or current BU, tuberculosis or leprosy

▪   Previous challenge with *M. ulcerans* JKD8049

▪   Resides in close proximity to endemic area (within 2 km) based on Victorian Department of Health epidemiologic data

▪   Family member/co-habiting with someone with a history of BU

▪   Previous history or examination finding consistent with *M. bovis* BCG vaccination

▪   Latent tuberculosis or chronic active hepatitis B or hepatitis C

▪   Vision impairment precluding self-examination of challenge site (and/or unable to use alternative to soft contact lenses)

▪   Intolerance of percutaneous injection

▪   Concurrent enrolment in a study which uses an investigational product or collects participant’s blood

▪   Venous access deemed inadequate for the phlebotomy demands of the study

▪   Any condition, including medical and psychiatric conditions that in the opinion of the Investigator, might interfere with the safety of the volunteer and/or study objectives

^*^ Clinical significance will be at the discretion of the Study Investigator.

## Schedule of events and procedures

The trial will be divided into four distinct periods: *screening, challenge, treatment,* and *healing.* See ‘Schedule of events’ for an example of the expected course of events and sampling.

### 1. Screening period

***Informed consent***

Written informed consent will be required prior to participation. Prospective participants will be invited to discuss the study during a facilitated meeting, which includes a brief presentation, which may occur as a group discussion with study investigators. The risks of the trial will be described, and prospective participants will also be allowed to ask questions privately. They may consider their involvement for up to 28 days, to allow them adequate time to consider participation.

To assess capacity to provide consent, participants will be invited to complete a multiple-choice quiz to demonstrate their understanding of the study and to ensure researchers have communicated details of the study appropriately. Incorrect answers will be explained by the researcher, and participants will have the opportunity to repeat the quiz again. If the study team are satisfied that the participant is voluntarily offering to participate in the trial, they will be invited to provide written informed consent. Written informed consent will be obtained by the responsible clinician on the day of any other procedure, including punch biopsy and therapeutic excisional biopsy.

***Screening procedure***

Screening aims to select participants at low risk of disease or treatment related complications. During screening, a medically qualified trial investigator will check that the prospective participant meets all eligibility criteria (i.e., meets all inclusion criteria and no exclusion criteria). They will gather this data in accordance with Table 1. Participants found to have any exclusion criteria on history or examination will not proceed to have investigations performed. Should a previously unrecognised condition be identified during screening, the participant will be informed by a qualified medical practitioner, and referred to their general practitioner (GP) or specialist for further investigation and management as relevant.

**Table 1.** History, examination and investigation of candidate participants.

History	Examination	Investigations
Age and biological sex	Height (cm)	Full blood count and film
Medical history	Weight (kg)	Urea, electrolytes, creatinine
Obstetric history and planning	BMI (kg/m ^2^)	Calcium, magnesium, phosphate
History of poor wound healing	Resting observations: - Heart rate - Blood pressure - Respiratory rate - Oxygen saturation - Body temperature (tympanic)	Liver function test panel and coagulation studies
History of excessive scarring		C-reactive protein
Smoking history		Erythrocyte sedimentation rate
Alcohol use history		HIV 1 / 2 Ag / Ab HTLV-1 Ab
Recreational substance use		Hepatitis B sAb, sAg, cAb
Psychiatric history	BCG vaccine scar (deltoid)	Hepatitis C Ab
Occupational history	Exposed skin check	TB-IGRA (QuantiFERON Gold)
Travel history	Cardiorespiratory examination	Peripheral lymphocyte subsets
Prescription medicine use	Gastrointestinal examination	Immunoglobulin quantification
Non-prescription medication use	Fitzpatrick skin type	HbA1c
Allergies (including antibiotic and anaesthetic allergy)	Upper limb physical examination	Electrocardiogram (ECG)
*M. bovis* BCG vaccine history	Visual acuity test (Snellen)	Audiometry testing
Tetanus vaccine history	Haematological examination	Urinary bHCG *(as relevant)*

***Medical history***

The initial clinic visit will include a detailed medical history to ensure that participants are healthy and at low risk of complications, including obtaining any concurrent medical conditions, medications (including non-prescription and recreational drug use), smoking history, alcohol consumption, allergies, and vaccination history. History will also discuss pregnancy and planning for pregnancy, in addition to the ability to use acceptable methods of contraception.

***Physical examination***

The initial clinic visit will include a targeted clinical examination including recording vital signs, weight and height to calculate body-mass index (BMI). Criteria will exclude volunteers with a high BMI, which is a reported risk factor for relapse ^36^. A skin check will document Fitzpatrick skin phototype ^34, 35^, and inspect for any evidence of previous or current BU or BCG vaccination. An examination of the upper limb will aim to identify any pre-existing limb abnormality or vascular insufficiency. A cardiorespiratory, gastrointestinal and haematological examination will be performed to evaluate for previously unrecognised medical conditions, with particular attention to potential cardiac and hepatic disease.

***Fitzpatrick skin phototype***

Participants with a Fitzpatrick skin phototype ≥ 5 are at higher risk of scarring ^34, 35^. Nevertheless, their inclusion has important implications for understanding BU in people of diverse backgrounds. They will therefore require an additional element of informed consent to participate, bearing this additional increased risk in mind.

***Investigations***

Volunteers will be screened for primary and secondary immunodeficiency; HbA1c may be used to exclude diabetes, which is a known risk factor for BU ^7^, including oedematous lesions ^25^, and may impair wound healing. Other investigations will include serology for retroviruses, and screening for cellular and humoral dysfunction. Infections that may increase the risk of hepatotoxicity (hepatitis B and C) will also be tested. Screening for latent tuberculosis with QuantiFERON-TB Gold Plus will prevent inadequate treatment of latent (asymptomatic) infection; this also tests non-specific cell mediated activity by IFN-γ release to mitogen; individuals with anti-INF-γ autoantibodies may be at risk of more severe mycobacterial infection ^37^. Investigations will also target potential issues related to antibiotics, including abnormal baseline ECG, electrolyte disorders (to reduce risk of prolonged QTc interval), hearing impairment, and pre-existing liver disease.

***Sampling***

Sampling throughout the trial will include blood collection (maximum 450 mL during any 3 month period) for laboratory safety analyses (LSA; see Table 2), microbiological and immunological analyses (see ‘Exploratory analyses’), including serum and peripheral blood mononuclear cells (PBMCs) to understand host responses to infection. Blood will be collected prior to ‘sham’ challenge and prior to subsequent JKD8049 challenge, and at the prespecified timepoints described under ‘Study procedure’ during the remainder of the trial.

**Table 2.** Laboratory safety assessments.

Panel	Parameter	
Haematology	Haemoglobin	Neutrophils absolute and %
	Haematocrit	Lymphocytes absolute and %
	Platelet count	Monocytes absolute and %
	Red blood cell count	Eosinophils absolute and %
	White blood cell count	Basophils absolute and %
Serum biochemistry	Alkaline phosphatase	Urea
	Alanine aminotransferase	Sodium
	Aspartate aminotransferase	Potassium
	Gamma-glutamyl transferase	Creatinine
	Total bilirubin	C-reactive protein
	Albumin	
	Total protein	
Coagulation	International normalised ratio	
	Fibrinogen	
	Activated partial thromboplastin time	

***Response to excipients and monitoring***

After signed consent is obtained and all eligibility criteria are met, the participant will be monitored as an outpatient (day-stay) to enable a ‘sham’ challenge in the contralateral forearm, to evaluate their response to the cryopreservative and excipients in the media; this also establishes if scarring occurs due to the injection itself, and that no other local skin reaction (e.g., dermatofibroma) develops. Participants will be observed for 4 hours, with observations every 10 minutes for 1 hour, then half-hourly thereafter. The ‘sham’ challenge will be performed in the same conditions as the subsequent challenge (see ‘Study setting’). Participants will be required to record a virtual diary using a secure RedCAP platform throughout the study, and all participant-recorded photographs will be uploaded to this platform. They will be asked to photograph the ‘sham’ challenge site daily for 3 days, and the site will be examined at each subsequent face-to-face visit (see ‘Schedule of visits’). Participants will be instructed to hold the camera 15 – 20 cm from the challenge site, in a well-lit environment, using flash if available. Participants will be provided with a paper tape measure to record the size of any lesion or reaction. Questionnaires in the participant diary will evaluate symptoms and tolerability of procedures using binary outcomes or Likert scales, as appropriate.

## 2. Challenge period

### Study setting

This single centre study will be conducted at Doherty Clinical Trials (DCT) in Melbourne, Victoria, Australia. This facility was established to facilitate the establishment of human challenge trials, and is supported by clinicians with experience in this field of research. The centre includes dedicated inpatient and outpatient clinical care areas, a pharmaceutical preparation area and access to qualified medical personnel. Challenge will be performed in a dedicated space within the trial facility, with personal protective equipment observing ‘contact’ precautions, including protective eyewear in case of accidental splash. Participants will be monitored as outpatients for 4 hours after challenge. Due to the long incubation period (4 – 5 months in Victoria, Australia, maximum 9 months ^9, 10^), and the long duration required for follow-up, all participants will be followed up as outpatients at the DCT centre.

### Challenge strain manufacture and cell banking

The proposed challenge agent, *M. ulcerans* JKD8049, has been extensively characterised for the purposes of human challenge ^38^. It is a fully antibiotic-susceptible, non-genetically modified Australian isolate, collected from a middle-aged male with a typical BU over their posterior calf, acquired in Point Lonsdale, Victoria, Australia. JKD8049 encodes all reported candidate vaccination antigens, and *M. bovis* BCG vaccination offers protection from disease in a murine mouse model using realistically low-doses of this *M. ulcerans* strain ^39^. *M. ulcerans* JKD8049 culture will occur in a secure facility following the principles of Good Manufacturing Practice. In brief, a library stock of JKD8049 will be serially passaged using animal-free media without chemical modification, with at least one clonal purification prior to the creation of a master cell bank. This master cell bank will be analysed for purity, potency and identity, including whole genome sequencing, as described previously ^38^. The working cell bank will consist of multiple, single-use, homogenous suspensions of *M. ulcerans* JKD8049 stored in an inert cryopreservative, glycerol, which is unlikely to produce any clinically meaningful adverse reaction at low volume (0.1 mL) and concentration ≤ 20% (v/v) ^40^. Purity, potency and identity testing of 10% of working cell bank vials will be performed after cryopreservation. The CFU count from these quality control cryovials will be used to calculate the dilution factor required to establish the final dose for injection in pH-neutral phosphate-buffered isotonic saline (PBS) as the diluent. PBS is a well-tolerated excipient ^41^, already used in the delivery of some vaccines. Previous studies ^38^ have demonstrated that the challenge agent is stable on ice for 4 hours with no significant loss of viability. The agent will be stored at -80°C and a cold chain will be established.

### Dosing of challenge strain

**Figure 2.**
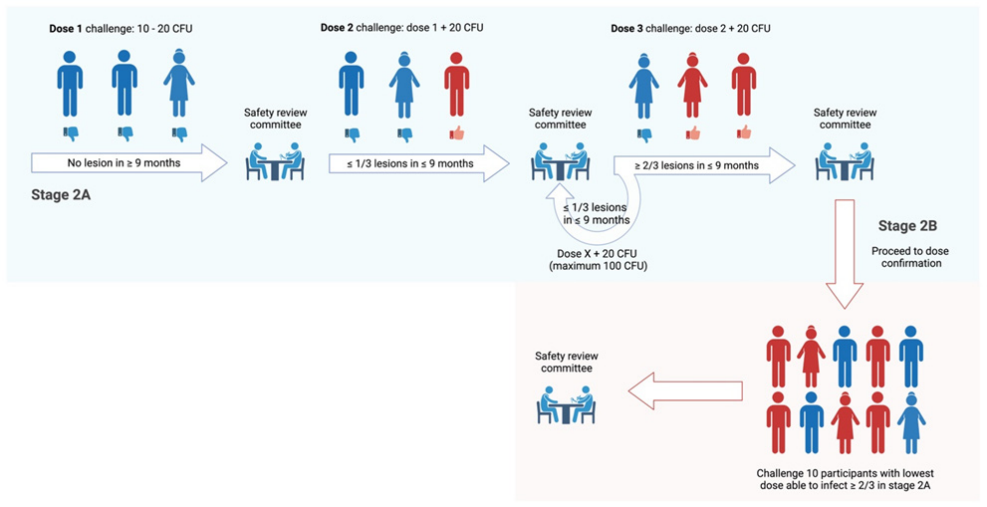
Dose escalation procedure from stage 2A (light blue) to 2B (light red). Created with BioRender.com.

Based on observations of a similar incubation period as mice, the infectious dose in humans is anticipated to also be very low ^42, 43^. First-in-human challenge will begin with a dose of 10 – 20 CFU, as doses in this range have previously demonstrated an attack rate of 100% in murine models using the same strain, prepared using identical methodology ^22^. Recruitment of additional participants for dose-escalation will occur no sooner than 9 months after first-in-human challenge fails to establish infection, as this is the maximum reported incubation period ^10^ ( Figure 2). Following review by the Safety Review Committee (see ‘Safety reporting’), subsequent dose-escalation will increase the CFU received per participant by 20 CFU (to maximum 100 CFU). Each increment will challenge three participants (Stage 2A), and if ≥ 2 of 3 are successfully challenged, then this dose will be used to challenge 10 subsequent volunteers in a dose confirmation study (Stage 2B). New participants will be recruited for dose confirmation, to avoid the impact on the host’s immune response following prior *M. ulcerans* exposure.

### Administration

If required, hair removal overlying the inoculation site will allow dressing adherence and improved visualisation for monitoring. The skin will be disinfected with a 70% alcohol wipe and allowed to air dry for 30 seconds. After thawing the cryopreserved working cell bank (on ice), the vial will be vortexed on low speed for 10 seconds. The *M. ulcerans* JKD8049 suspension will be diluted to the required dose using PBS in a low dead-space syringe. A maximum volume of 0.1 mL will be injected, by trained study staff, at approx. 45° angle into the subcutaneous tissue using a sterile, thin-walled 30-gauge low dead-space needle, at a depth of 2 – 3 mm, approximating the length of a mosquito’s proboscis ^44, 45^. The skin will be ‘pinched’ to aid subcutaneous injection. If feasible, reproducibility may be standardised using a fabricated luer-lock cap, manufactured to guide the challenge material ^46^. With the bevel of the needle facing up, the needle will be slowly aspirated prior to injection, to ensure no inadvertent intravascular administration. The material will then be injected slowly over ~ 10 seconds. Following injection, a cotton swab will be used to apply gentle pressure over the injection site as the needle is withdrawn, to minimise reflux of the challenge material. Simple bandaging without antiseptic will be used to cover the inoculation site. Simple analgesia (paracetamol 1 g orally) will be offered for pain if there are no contraindications. Prior to discharge, participants will be instructed on the possible lesion appearance, with take-home visual instructions and images. Participants will also be instructed on how to perform photography of the challenge site and how to navigate the online portal to upload images and clinical information.

### Monitoring after challenge

Following challenge, participants will complete their participant diary daily for 3 days, including self-collected serial photography of the challenge site. Thereafter, virtual monitoring during this period will include twice weekly participant diary entry. In-person review for physical examination, LSA and exploratory immunological and microbiological analyses will occur 3, 7 and 14 days after challenge, and monthly thereafter (see ‘Schedule of events’). Participants will otherwise be asked to examine the challenge site daily to monitor for any lesion. If any visible lesion develops, they will be instructed to notify a trial investigator for face-to-face review. The development of a lesion bookmarks the end of the ‘challenge’ period.

## 3. Diagnosis and treatment period

### Case definition

The appearance of a nodule, plaque, papule, localised induration, erythema, generalised oedema or ulceration, at or in proximity to the challenge site, will be classified as a ‘probable’ case. A combined clinical and microbiological case definition will define a confirmed BU; the presence of IS *2404* DNA by PCR (either by swab or tissue diagnosis via biopsy) is confirmatory using cycle threshold defined as ≤ 40 cycles. This is the current ‘gold standard’ diagnostic tool with 100% specificity for Australian clinical isolates ^47^.

### Expected outcome

If a participant reports a lesion, they will be reviewed by the trial team within 48 – 72 hours. The expected outcome is that an ‘early lesion’ (patch of erythema and/or induration) will develop into a ‘pre-ulcerative lesion’ (nodule/plaque/pustule); in the event that they develop an ‘early lesion’ or ‘pre-ulcerative lesion’, the participant will be asked to monitor the lesion and return for review if ulceration occurs. If any ‘early lesion’ fails to progress into a pre-ulcerative lesion despite ≥ 10 days of monitoring, treatment will be initiated. If a ‘pre-ulcerative’ lesion persists after 7 days without progressing to ulceration, treatment will be initiated to minimise subcutaneous (subclinical) advancement of infection (see ‘Study procedure’ for further detail, and the procedures for other outcomes that are less likely to occur). Follow-up frequency will increase to weekly for 4 weeks after any lesion is reported.

### Antibiotic treatment

The WHO recommended antibiotic regimen is oral rifampicin (10mg/kg, maximum 600 mg, once daily) and clarithromycin (7.5mg/kg, maximum 500 mg, twice daily) for 8 weeks, following the results of a randomised trial that demonstrated all-oral therapy was non-inferior to injection antibiotic therapy and cured 96% of participants with early, limited BU ^20^. Notably, the majority of those who had an unsuccessful outcome reported in this trial were lost to follow up or did not adhere to per-protocol wound care. Relapse risk (~1%) will be further minimised by selecting volunteers without risk factors for relapse, including immunocompromise and high BMI ^36^. In Australia, observational evidence suggests that 6 weeks of antibiotic therapy is likely to be as effective as 8 weeks of therapy in select patients, with 100% of small lesions successfully treated with 6 weeks of antibiotic therapy ^48^; this is supported by studies demonstrating culture clearance within 20 days of treatment in all samples analysed ^49^. Participants will be prescribed antibiotic treatment according to the schedule listed in ‘Study procedures.’ They will be provided with an information pamphlet on side effects, when and how frequently to take the medication, and will be asked to report all side effects via their participant diary (see ‘Risk assessment’ for further detail). A dosette box with each day of the week clearly labelled will be supplied to support participants with the antibiotic schedule and to monitor adherence. In the unlikely event that participants relapse, a repeat course of antibiotics, typically rifampicin and a fluoroquinolone, with or without surgical excision of the lesion, will be administered.

### Wound care

All lesions/wounds will be reviewed by an experienced clinician to ensure appropriate dressings are applied and the frequency of dressing changes is optimised (typically alternate daily, depending on exudate volume). Participants should be able to manage their own dressing changes, with individualised training on aseptic technique, an ample supply of dressing equipment, and instructions to avoid other topical products. Written instructions and telephone contact details of study investigators will be provided to participants in case of unexpected wound deterioration. Eligibility criteria will include participants who are up-to-date with tetanus vaccination, as wounds with tissue damage are classified as tetanus-prone ^31^.

All wounds healing by secondary intention will be dressed appropriately with an absorbent dressing. For open wounds, topical preparations such as Flaminal ^50^ may be employed as an adjunct to wound dressings; these allow the base of the wound to remain hydrated, while debriding agents continuously dissolve necrotic tissue, and contain antibacterial properties to minimise secondary bacterial infection. Dressings for open wounds aim to minimise environmental exposure and secondary bacterial infection. Participants will also be instructed to minimise trauma to the wound, as this may exacerbate inflammation and wound breakdown. To minimise the impact of scarring, participants will be provided with a hypoallergenic moisturiser (with sun protection) to aid scar healing ^51^.

Secondary bacterial infection is infrequently documented ^52^ but should be considered if the wound demonstrates clinical features of pain and/or acute inflammation. In this event, a clinician will perform a history evaluating for systemic features of infection (e.g., fevers, rigors) and examine the participant, including vital signs, wound assessment for signs of superinfection and locoregional spread (such as lymphangitis). Additional investigations will be performed as clinically appropriate (e.g., wound and blood cultures, tissue biopsy for histopathology) and if secondary bacterial infection is likely, the clinician will prescribe appropriate antibiotic treatment with minimal risk of drug interaction or hepatotoxicity.

### Surgical treatment

Surgery is not usually required in the treatment of BU but has a role in allowing avoidance of, or significantly shortening the duration of, antibiotic treatment ^53, 54^. In this trial, participants will be given the option to have the lesion excised surgically with primary closure, reducing the duration of antibiotics required. Australian observational evidence suggests that 14 – 28 days of therapy is adequate to cure those who receive antibiotics and surgery ^53^. We propose using a duration of 4 weeks if the tissue margins are involved (by inflammation and/or AFBs), or 2 – 4 weeks if the tissue margins are uninvolved, guided by the participants’ tolerability of antibiotics. The duration of 4 weeks if margins are involved is selected based on evidence suggesting that organism sterilisation is achieved following 28 days of treatment in murine ^55^ and human tissue ^49, 56^, although culture positivity does not necessarily correlate with relapse ^57^. As the residual organism burden is expected to be very low in these scenarios, this duration balances the risks of an abbreviated duration of antibiotics with the low risk of relapse. The involvement of tissue margins is strongly associated with risk of relapse after surgery, nevertheless, 5 of 37 (13.5%) of patients in a prior study relapsed despite negative margins ^58^, so a brief duration of antibiotics (≥ 14 days) will still be required to further minimise relapse risk. Participants who demonstrate antibiotic intolerance (at any stage of treatment) will also have the option of surgical excision and primary closure. Surgical excision is anticipated to leave a linear scar. Primary closure will be performed by an experienced plastic surgeon under local anaesthetic. In a cutaneous human leishmaniasis model of infection, focus group research suggested that therapeutic excision was the favoured option, allowing researchers additional tissue for analysis, in addition to psychologically reassuring participants that the infection was ‘removed’ ^19^; focus groups will also explore whether this is a preferred option in the proposed MuCHIM study.

### Lesion sampling

In the case of ulcerated lesions, dry swabs will be performed to confirm the presence of *M. ulcerans* DNA within the lesion using IS *2404* PCR. Any undermined wound edge will be swabbed, ensuring material is visible on the tip of the swab; if this is negative, a 3 mm punch biopsy will be performed on the edge of the lesion ^59^. For non-ulcerative lesions, a minimally-invasive biopsy device ^60, 61^ will be used to test the presence of *M. ulcerans* DNA. This biopsy is not expected to leave a scar, as the wound created is just 0.21 mm in diameter, and also therefore does not require local anaesthesia ^60^. In addition, a 3 mm punch biopsy tissue sample will also be performed for IS *2404* PCR confirmation, which will provide a comparison to the minimally-invasive test. As the minimally invasive biopsy will be followed immediately by a punch biopsy, local anaesthetic will be injected prior to the minimally invasive biopsy sample, although if non-inferior, future applications of the trial will avoid the need for local anaesthetic by using only the minimally invasive device. For participants who elect to have the lesion excised, the tissue will be processed for immunological analyses. All participants who do not undergo a therapeutic excision will be invited to have an additional 4 mm punch biopsy collected at the time of diagnostic sampling (see ‘Exploratory analyses’).

### Monitoring

Once antibiotic treatment is initiated, ‘active surveillance’ for side effects will include weekly adverse reaction screening (in person or telephone call) while participants are taking antibiotics, and reflex examination and investigation by a qualified physician in the event that adverse reactions are reported. Blood sampling for LSA and exploratory analyses will be performed at baseline (before antibiotics) and weekly for 4 weeks, then 2-weekly thereafter for a further 8 weeks. ECG will be performed at baseline and 1 – 2 weeks into antibiotic therapy to assess for QTc prolongation (i.e., after antibiotic steady state is reached). Participants will also complete the participant diary twice weekly, including reporting of any antibiotic side effects. Participants will be encouraged to report any symptom, and grade their severity in terms of function and impact on their activities of daily living using the participant diary. Regular face-to-face outpatient follow-up will enable prompt clinical evaluation, initiation of treatment and wound care as required. A detailed appraisal of antibiotic side effects is described in ‘Risk assessment.’ For an exploratory analysis, participants may also be invited to provide a faecal microbiome sample and skin swabs for analysis prior to, during and after the completion of antibiotic therapy.

After a lesion is first reported, participants will complete the Dermatology Life Quality Index (DLQI) questionnaire ^62^, which is used to measure the impact of skin disease on their quality of life. Participants will be invited to complete this weekly for 1 month after the lesion is first reported, then monthly until study completion. The Generalised Anxiety Disorder 7 score (GAD-7) ^63^ will also be used to measure mood, beginning at the time of challenge and continuing monthly until a lesion is reported; the questionnaire will then be performed at the same intervals as the DLQI.

### Schedule of visits and procedures

**Figure 3.1.**
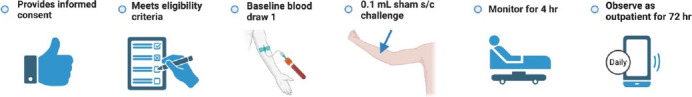
Screening.

A detailed schedule of visits and procedures is summarised in Table 3 (‘Visit schedule’) for the most likely (expected) outcome. A schematic summary of expected and unlikely outcomes is presented in ‘Study procedures’ ( Figure 3.1– Figure 3.4.2). 

**Table 3A.** Schedule of events; screening and challenge periods.

	Screening period					Challenge period				Month 1								Monthly until leslon reported							
			Mon	Tues	Wed	Thur	Fri	Sat	Sun	Week 1 Mon/Thu		Week 2 Mon/Thu		Week 3 Mon/Thu		Week 4 Mon/Thu		Week 1 Mon/Thu		Week 2 Mon/Thu		Week 3 Mon/Thu		Week 4 Mon/Thu	
Study visit	1	2	3	4	5	6	7	8	9	10	11	12	13	14	15	16	17								
Study day		-1 to -28	0	1	2	3	4	5	6	7	10	14	17	21	24	28	31								
Time window (+/- days)			0	0	0	0	1	1	1	2	2	2	2	2	2	2	2	2	2	2	2	2	2	2	2
Eligibility check	X																								
Written consent		X																							
Verbal consent			X			X															X				
History		X																							
Examination		X				X															X				
Vital signs		X	X			X															X				
Screening investigations		X																							
12-lead ECG		X				X																			
Urinary bHCG (as appropriate)		X	X			X																			
Laboratory safety assessment			X			X				X	X		X					X	X		X				
Excipient-only challenge			X																						
*M. ulcerans* JKD8049 challenge						X																			
AE screening			X	X		X	X														X				
Virtual volunteer diary				X	X	X	X	X	X	X	X	X	X	X	X	X	X	X	X	X	X	X	X	X	X
Self-collected photograph				X	X	X	X	X	X	X	X	X	X	X	X	X	X	X	X	X	X	X	X	X	X
PBMC collection			X			X				X	X		X					X	X		X				
Serum collection			X			X				X	X		X					X	X		X				
Microbiology blood sampling						X				X	X		X								X				
Skin swabs						X																			
GAD-7 questionnaire						X							X								X				
Time (min)	20	60	300	20	20	300	20	20	20	10	10	10	10	10	10	10	60	10	10	10	10	10	10	10	60

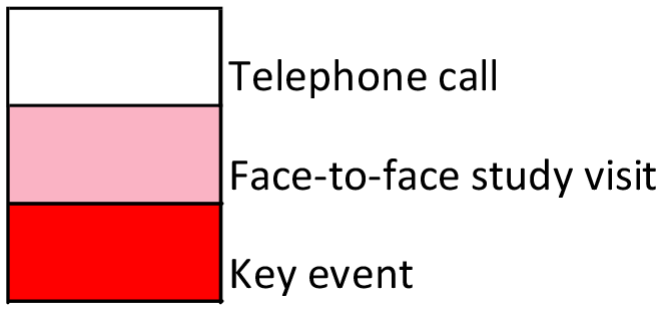

**Table 3B.** Schedule of events; treatment period.

	Treatment Period																							
	Month 1 - weeekly visits								Month 2 - fornightly visits								Month 3 - fornightly visits							
	Week 1 Mon/Thu		Week 2 Mon/Thu		Week 3 Mon/Thu		Week 4 Mon/Thu		Week 1 Mon/Thu		Week 2 Mon/Thu		Week 3 Mon/Thu		Week 4 Mon/Thu		Week 1 Mon/Thu		Week 2 Mon/Thu		Week 3 Mon/Thu		Week 4 Mon/Thu	
Time window (+/- days)	2	2	2	2	2	2	2	2	3	3	3	3	3	3	3	3	3	3	3	3	3	3	3	3
Verbal consent	X		X		X		X				X				X				X				X	
Examination	X		X		X		X				X				X				X				X	
Vital signs	X		X		X		X				X				X				X				X	
AE screening	X		X		X		X				X				X				X				X	
Urinary bHCG (as appropriate)	X																							
12-lead ECG	X						X																	
Laboratory safety assessments	X		X		X		X				X				X				X				X	
Diagnostic sampling				X																				
Treatment initiation					X																			
Vitual volunteer diary	X	X	X	X	X	X	X	X	X	X	X	X	X	X	X	X	X	X	X	X	X	X	X	X
Self-collected photograph	X	X	X	X	X	X	X	X	X	X	X	X	X	X	X	X	X	X	X	X	X	X	X	X
PBMC collection	X		X		X		X				X				X				X				X	
Serum collection	X		X		X		X				X				X				X				X	
Microbiology blood sampling	X		X		X		X				X				X				X				X	
Faecal microbiome sampling	X						X								X								X	
Skin swabs	X						X				X				X				X				X	
DLQI and GAD-7 questionnaires	X		X		X		X		X								X							
Time (min)	120	10	60	10	240	10	60	10	15	10	60	10	15	10	60	10	15	10	60	10	15	10	60	10

**Table 3C.** Schedule of events; healing period.

	Healing period															
	Monthly for 3 months								Monthly for 3 months ( *scar maturation - virtual follow up only*)							
	Week 1 Mon/Thu		Week 2 Mon/Thu		Week 3 Mon/Thu		Week 4 Mon/Thu		Week 1 Mon/Thu		Week 2 Mon/Thu		Week 3 Mon/Thu		Week 4 Mon/Thu	
Time windows (+/- days)	5	5	5	5	5	5	5	5	5	5	5	5	5	5	5	5
Verbal consent								X								
Examination								X								
Vital signs								X								
AE screening								X								
Laboratory safety assessment								X								
Virtual volunteer diary	X	X	X	X	X	X	X	X	X				X			
Self-collected photograph	X	X	X	X	X	X	X	X	X				X			
PBMC collection								X								
Serum collection								X								
Skin swabs *								X *								
Faecel microbiome sample *								X *								
DLQI and GAD-7 questionnaires	X								X							
Time (min)	10	10	10	10	10	10	10	60	10				10			

* Faecal microbiome sample and skin swabs only required at the final face-to-face visit

### Study procedures

**Figure 3.2.**
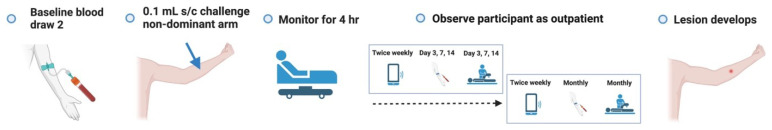
Challenge.

**Figure 3.3.1.**
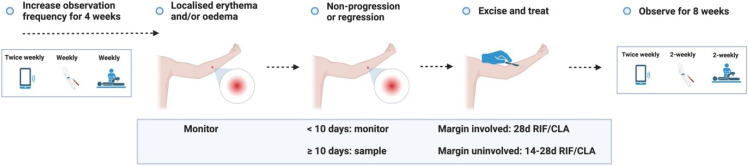
Treatment – Expected outcome 1A: Therapeutic surgical excision of early lesion.

**Figure 3.3.2.**
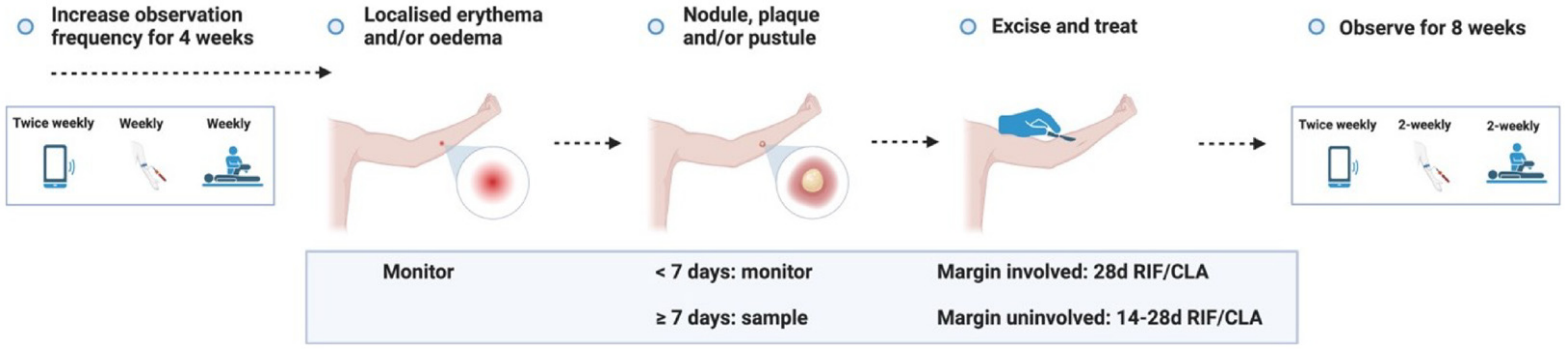
Treatment – Expected outcome 1B: Therapeutic surgical excision of pre-ulcerative lesion.

**Figure 3.3.3.**
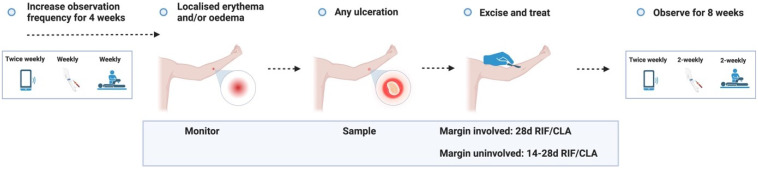
Treatment – Expected outcome 1C: Therapeutic surgical excision of ulcer.

**Figure 3.3.4.**
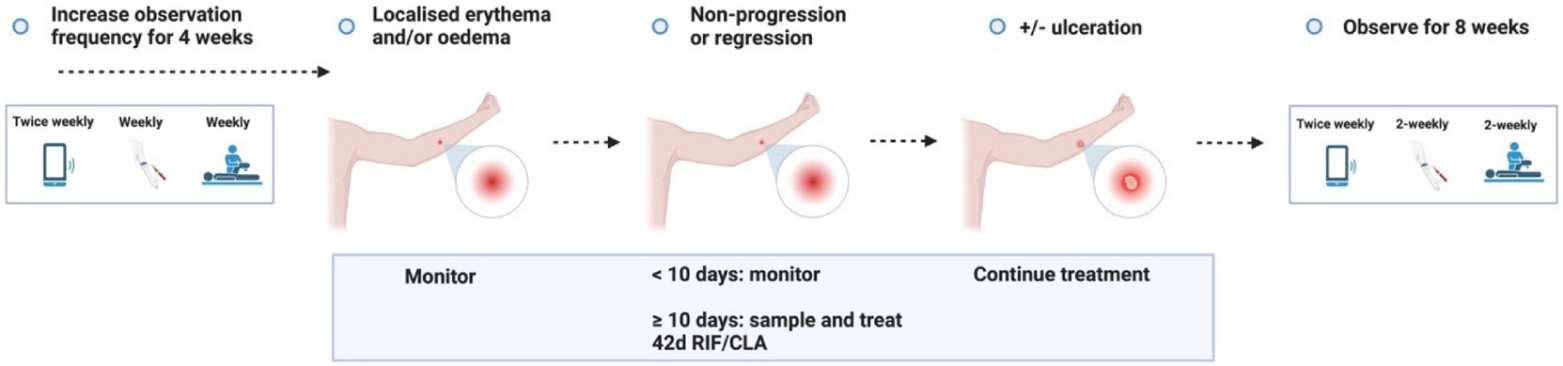
Treatment – Expected outcome 2A: Antibiotic treatment without surgery.

**Figure 3.3.5.**
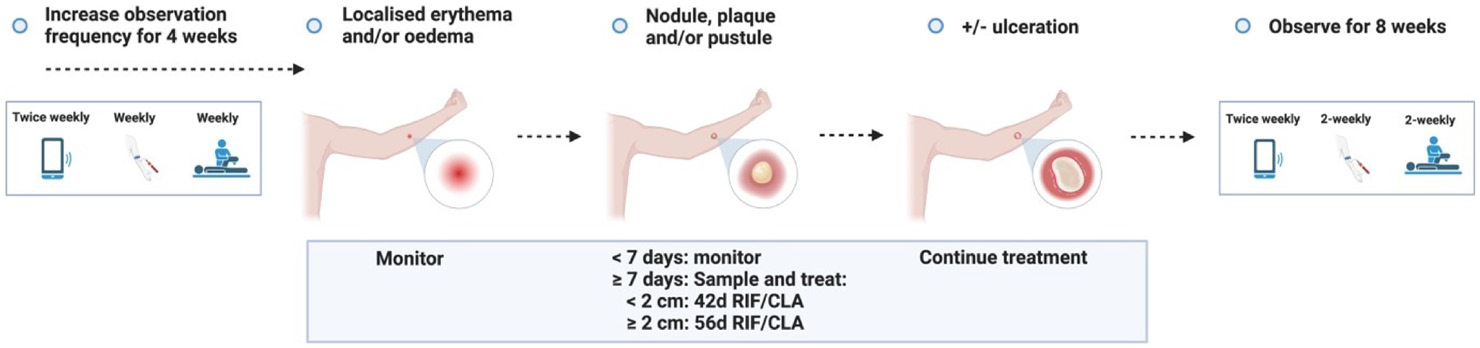
Treatment – Expected outcome 2B: Antibiotic treatment without surgery.

The 2 cm threshold (measured from the indurated edge) is based on Australian observations that small lesions are cured with 6 weeks of treatment (most ≤ 400 mm ^2^) ^48^ which is now local practice in some high-caseload settings.

**Figure 3.3.6.**
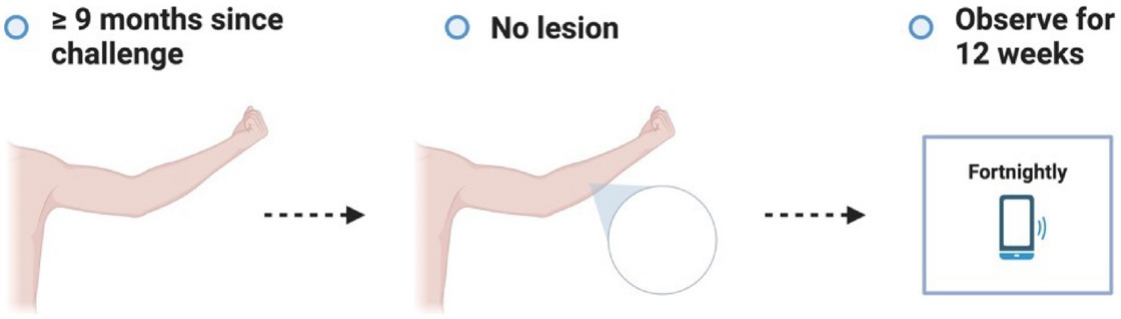
Treatment – Alternative outcome: No lesion at 9 months.

These participants will no longer be eligible to participate in a subsequent dose escalation study. They will all be asked to contact trial investigators in the unlikely event that a lesion develops after study completion.

**Figure 3.3.7.**
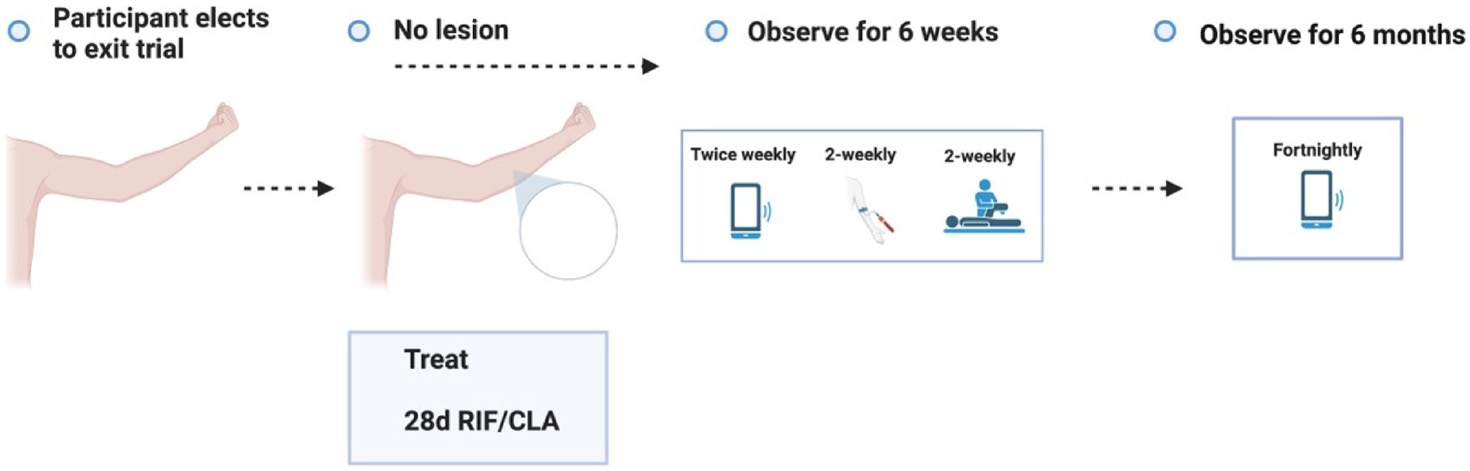
Treatment – Unexpected outcome: No lesion, participants exits trial prematurely.

Participants who meet the STOP criteria will be offered pre-emptive treatment, and a frequent follow up period of 6 weeks will be offered to ensure antibiotic compliance, no adverse antibiotic reactions and no paradoxical reactions. The participant will be followed up using the least restrictive method thereafter if the above plan is unable to be observed (e.g., telephone, email) and will be linked in with their usual GP.

**Figure 3.3.8.**
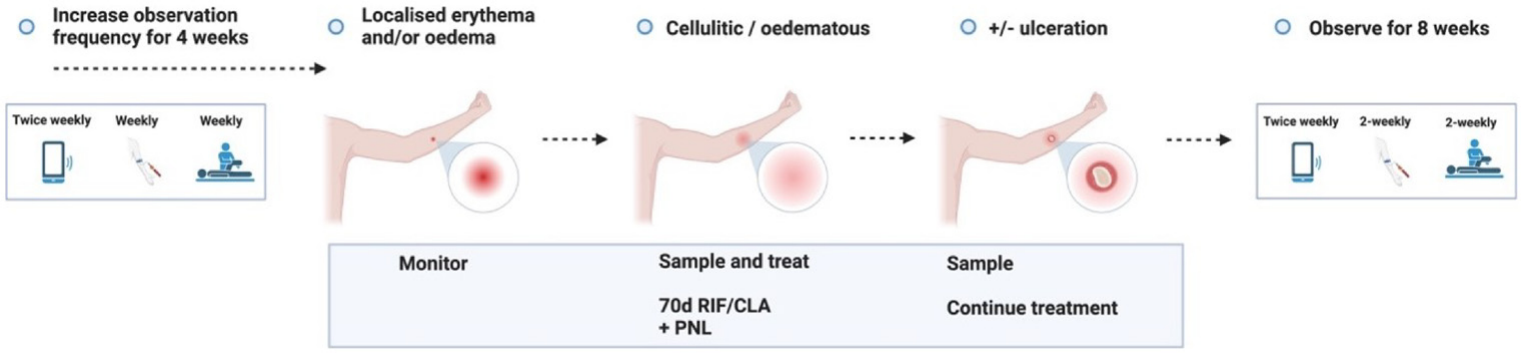
Treatment – Unlikely (adverse) outcome: Cellulitic / oedematous lesion.

Cellulitic/oedematous lesions will be defined as erythema and/or oedema ≥ 5 cm (in maximum diameter) at the challenge site ≤ 7 days from when the lesion is first reported. Urgent clinician review (within 24 hours) will also evaluate and consider treatment of superimposed non- *M. ulcerans* skin/soft tissue infection.

**Figure 3.4.1.**
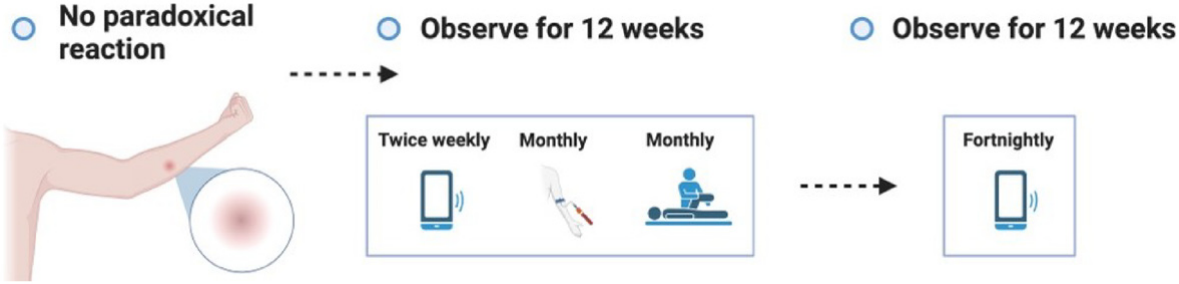
Healing – Expected outcome.

**Figure 3.4.2.**
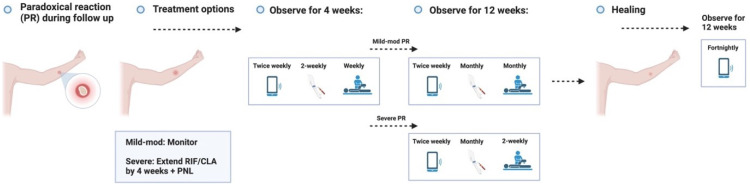
Healing – Unlikely outcome: Paradoxical reaction.

### Study end points

The study end point will be reached when:

1. An ‘early lesion’ (erythema, and/or induration at the challenge site) has been present without progression to pre-ulceration, or spontaneously regresses, within ≥ 10 days,

2. Any pre-ulcerative lesion has been present for ≥ 7 days, including nodules, plaques and pustules, but excluding cellulitic/oedematous lesions,

3. There is any sign of ulceration

4. No lesion develops after 9 months of follow-up

5. Participant meets STOP criteria

## 4. Healing period

The beginning of the healing period is defined as 12 weeks after a lesion is first noted, although healing of the lesion is anticipated to begin at some stage during antibiotic therapy. This period is anticipated to include ongoing wound healing after the completion of antibiotics and scar maturation. In-person monitoring during this period will occur 4-weekly for 12 weeks, including blood sampling for LSA and exploratory analyses, and twice weekly participant diary entry. After the final face-to-face visit, their patient diary alone will be used for routine follow-up for a further 12 weeks to document scar maturation.

### End of study

Stage 2A of the study will end if at least two participants are successfully challenged and when all participants complete their final study visit, as defined in ‘Study procedure.’ Each participants’ final in-person visit is anticipated to occur 9 – 10 months after recruitment (with an additional 12 weeks of infrequent virtual follow-up thereafter). This assumes an incubation period of approximately 3 – 4 months, as lesions are likely to be noted by participants sooner than may otherwise be reported in the field. Participants who have completed the trial will be provided with the contact details of medical clinics with experience managing BU, as well as the trial management team, in the unlikely event that they develop a lesion after the end of study. At study completion, all participants will complete an exit questionnaire, which will also inform future applications of the trial.

## Exploratory analyses

### Systemic immune responses

Blood will be collected in EDTA tubes over serial timepoints, with peripheral blood mononuclear cells (PBMCs) and plasma isolated using Ficoll density gradient centrifugation. Plasma supernatant will be collected, and aliquots will be stored at -80°C. The corresponding PBMCs will be resuspended in a cryopreservative (such as foetal calf serum-10% DMSO) and stored at -80°C. ELISA measurements will be performed on plasma samples to analyse a broad suite of inflammatory cytokine and chemokine concentrations using a commercially available multiplex panel, including immune signatures associated with disease ^15^. High-dimensional flow cytometry on PBMCs will determine changes in immune populations including innate cells (e.g., monocytes, dendritic cells) and adaptive lymphocytes (e.g. B cells and T cells) as well as their activation status. PBMCs may also be analysed for markers of cytokine release (e.g., ELISpot). Antibody responses to *M. ulcerans* antigens will also be explored ^64^.

### Tissue immune responses

For participants who elect to have the lesion surgically excised, the tissue sample may provide a rich source of information regarding immune responses to infection in the skin and subcutaneous tissue. Tissue harvested by therapeutic excision will be split; one sample will be fixed in formalin for histology and immunohistochemistry and the other will be dissociated into single cell suspensions stored at -80°C for immunological downstream analyses as outlined above. High-dimensional flow cytometry will be used to investigate tissue cell populations and activation status. For participants who prefer to have any lesion treated with antibiotics alone, they will be invited to have an additional 4 mm skin punch biopsy performed at the time of the diagnostic biopsy (or at the time of diagnostic swab in ulcerated lesions) for analysis. If adequate resources and samples are available, spatial transcriptomics may also be performed.

### Understanding the host microbiome over time

There is an increasing awareness of the importance of the host microbiome on health outcomes ^65^. If resources allow, this optional study procedure aims to understand the relative composition of microbial populations and dynamics over time. If participants are unwilling or unable to provide samples, it will have no bearing on subsequent participation in the study. Skin swabs may be collected during face-to-face assessment, and faecal microbiome samples may be collected using a dedicated self-collection kit, with samples stored in a stabilising agent and cryopreserved at -80°C for subsequent analysis.

### Microbiological features of infection

Evidence of bacterial dissemination has been observed in another natural host, the Australian ringtail possum, although this appears to be unsuccessful at establishing infection in organs ^66^. If resources allow, we aim to collect whole blood for IS *2404* PCR and mycobacterial culture. We also aim to routinely perform mycobacterial culture using fresh tissue not used for other purposes, which may enable testing of antibiotic susceptibility ^38^.

## Risk assessment

CHIMs can generally only be established in treatable or self-limiting diseases where irreversible pathology is unlikely to occur ^67^. This trial therefore balances the risks of disease and treatment with the potential benefit derived from successful implementation of the model.

### Antibiotics

A unique aspect of this trial is that the risk of antibiotic adverse events may be greater than the risks related to a small, early BU. Nevertheless, the risks related to antibiotic use are well characterised. Retrospective observational Australian evidence suggests that antibiotic complications are not uncommon, although risk factors are well established, including reduced renal function, highlighting the importance of participant selection ^5^. Recent evidence from a randomised trial of antibiotic regimens for BU demonstrated that of 146 participants, 6% of patients prescribed oral combination antibiotic therapy with rifampicin and clarithromycin experienced an adverse event, although none were defined as a serious adverse event. One participant experienced ototoxicity and two experienced non-severe QTc prolongation ^20^. Baseline screening and regular protocolised follow up monitoring, including active and passive surveillance strategies, will observe for side effects, and enable prompt intervention.

This trial uses evidence-based treatment to offer participants the shortest possible duration of effective treatment required to achieve cure. As antibiotic toxicity may persist during treatment, treating participants with the shortest duration will result in reduced risk to participants, less inconvenience and reduced costs of more prolonged antibiotics. It is anticipated that the majority of participants not undergoing therapeutic excision will be treated with 6 weeks of combination antibiotic therapy, aligned with contemporary local practice ^48, 49, 55^.

Common side effects from rifampicin and clarithromycin include nausea and reduced appetite, and dysgeusia due to clarithromycin. Participants will be encouraged to maintain adequate hydration, and antiemetic therapy (e.g., ondansetron 4 – 8 mg three times daily, as required) may be prescribed if there are no contraindications. Dividing rifampicin into two daily doses (i.e., 300 mg twice daily) may also improve symptoms in participants who experience treatment-related nausea. Red discoloration of bodily fluids is usually observed due to rifampicin, this is benign and resolves after treatment, but it may cause alarm if they are not warned in advance. Participants who use soft contact lenses should consider alternatives due to staining. Due to the risk of drug interactions with any trial treatment antibiotic, participants will be instructed to inform the trial team of any new medications or non-prescription therapies.

Transient mild elevations of liver enzymes may occur in people taking any of the antibiotics in this trial, although clinically significant hepatotoxicity is rare; it is not currently routine practice to monitor liver function in patients with BU on these antibiotics without pre-existing risk of liver injury or baseline abnormality, but will be checked weekly during treatment as an additional precaution. Of 3,280 participants in a trial of rifampicin for latent tuberculosis infection (LTBI), 31 participants (0.9%) developed a severe adverse reaction, the most common was hepatotoxicity in 11 (0.3%), followed by 6 (0.2%) with rash or drug allergy, and 6 (0.2%) with haematological adverse event; no deaths occurred due to rifampicin therapy in this study ^68^. Although the LTBI trial evaluated 4 months of rifampicin therapy, MuCHIM anticipates 2 – 6 weeks of therapy for most participants. Additionally, clinically apparent hepatitis is very uncommon in people prescribed clarithromycin (3.8 per 100,000 prescriptions) ^69^. Although clinically significant hepatitis due to these antibiotics is rare, it is further mitigated by ensuring that the participant does not consume alcohol or other hepatotoxins for the duration of treatment, and excluding pre-existing subclinical hepatitis prior to study inclusion. All participants will be instructed to stop antibiotic treatment and to notify the trial team if they develop right upper quadrant abdominal pain, persistent vomiting, or jaundice. Complete recovery of rifampicin and clarithromycin induced hepatitis is expected after stopping treatment ^69^.

Ototoxicity in the form of sensorineural hearing loss (SNHL) may be a rare complication of clarithromycin use. Most of the evidence on this potential association has been from case reports or series ^70^, and given its rarity, larger studies are required to confirm an association. Studies in Guinea pigs have found evidence of ototoxicity, which is reversible ^71^. Irreversible hearing loss attributed to clarithromycin appears to be very rare ^72^. In a systematic review of case reports and series, 70 of 78 patients with SNHL attributed to macrolides demonstrated reversal following macrolide discontinuation alone ^70^, usually 1–3 weeks after cessation ^73^. However, these studies are subject to numerous biases related to retrospective study methodology. A more recent prospective longitudinal study assessed the association of macrolide use and ototoxicity, with no observed association ^74^. A meta-analysis also could not demonstrate a relationship between macrolides and SNHL ^75^. Finally, a large database nested case-control study was also unable to demonstrate an association between macrolides and SNHL ^76^. Tinnitus is known to be associated with macrolide use, although the few case reports of this complication suggest it is reversible upon cession of the macrolide ^77^.

If either rifampicin or clarithromycin are unable to be continued, either may be replaced by ciprofloxacin (dosed at 500 mg orally, twice daily). This antibiotic may also cause QTc prolongation, so a baseline ECG to exclude congenital or acquired long QTc syndrome is also required; if clarithromycin and ciprofloxacin are used in combination, then additional monitoring for QTc prolongation is required with ECG performed weekly. Participants must be warned of tendinopathy and will be instructed to contact study investigators if this occurs, although risk factors (including age, diabetes and other comorbidities) are minimised by selection criteria ^78^. Ciprofloxacin and clarithromycin may cause gastrointestinal disturbance and diarrhoea; persistent diarrhoea (≥ 24 hours) will be tested for *Clostridioides difficile* infection, although clarithromycin appears to confer a comparatively lower risk than fluoroquinolones ^79^. Central nervous system symptoms such as agitation, restlessness, and confusion have been associated with clarithromycin ^80^ and fluoroquinolones ^81^, and peripheral neuropathy is a recognised but very rare side effect; a 28 day course of ciprofloxacin in people aged < 60 has a number needed to harm of 86,900 ^82^. Nevertheless, ciprofloxacin is an alternative only for participants who are unable to tolerate first-line treatment.

### Expected time to healing and paradoxical reactions

Spontaneous healing without treatment has been reported in a small number of immunocompetent patients ^83, 84^, although why some people mount successful immune responses to infection is unclear. Following antibiotic initiation, most early, limited lesions (≤ 2 cm diameter) heal after a median of 91 days ^85^. 3.8% of individuals (aged 15 – 60) with BU in Victoria’s Bellarine Peninsula have been reported to develop an acute oedematous form of BU ^25^, which may require pre-emptive treatment with corticosteroids ^86^. Paradoxical reactions are observed after a median of 39 days ^87^ in approximately one fifth of patients undergoing antibiotic treatment ^28, 87^. These are typically mild, and rarely require corticosteroids to blunt the immunological response ^88, 89^ with prolongation of their antibiotic treatment in selected cases ^87^. As paradoxical reactions give the impression of wound deterioration despite appropriate therapy, participants will be informed of this possibility prior to commencing treatment. Nevertheless, these reactions are likely to be less common, and also unlikely to be severe in small lesions treated early in healthy young adults. Surgical intervention remains an option for participants who are unable to complete the full duration of antibiotic treatment or who prefer it for aesthetic purposes, which will reduce the time to healing ^85^.

Although unlikely, prednisolone will be offered to participants with severe paradoxical reactions and cellulitic/oedematous disease. This will be managed by experienced Infectious Diseases physicians. The dose will be 0.5 mg/kg for 1 – 2 weeks, then tapered to a maximum duration of 6 – 8 weeks ^90^. A proton pump inhibitor without RIF/CLA drug interaction, pantoprazole 20 mg, will be co-prescribed to minimise symptoms of gastritis when prednisolone doses of ≥ 20 mg per day are prescribed. 2-weekly blood sampling will include electrolyte and blood glucose monitoring during prednisolone treatment. Participants will also be asked to monitor for additional symptoms of short-term prednisolone use, including mood disturbance (specifically agitation and/or elevated mood), sleep disturbance, increased appetite, fluid retention, and gastrointestinal discomfort. Examination during this treatment will include observations, with particular attention to hypertension and weight gain. Finally, all participants who are commenced on corticosteroids will be screened for latent strongyloidiasis prior to initiation ^91^; if positive or equivocal, empirical treatment will minimise reactivation risk in the unlikely event that corticosteroids are needed.

### Spread to local structures

Restricted by a low and narrow optimal growth temperature, *M. ulcerans* is unable to successfully establish infection in visceral organs, and therefore preferentially establishes infection in more superficial locations ^92^. Contiguous spread to local structures, such as nearby bone or joint, is rare in the Australian context ^26^, and is unlikely to occur in lesions that are treated soon after recognition. As an additional safety measure, all participants who develop a lesion will be treated with antibiotics to which the challenge organism is susceptible *in vitro,* even if lesions are fully excised, in order to target organisms which may have spread subclinically.

### Phlebotomy

The total blood volume taken at each visit will be approx. 30 mL. The total volume of blood collected over each 3 month period will be a maximum of 450 mL. This volume should not compromise otherwise healthy participants. Risks associated with venepuncture include pain and bruising at the site of venepuncture, pre-syncope and syncope, which is mitigated by selection criteria which exclude volunteers with intolerance to percutaneous intervention, and ensuring participants are well hydrated prior to all percutaneous interventions.

### Punch biopsy

To confirm the presence of *M. ulcerans* via IS *2404* PCR in non-ulcerative lesions, minimally invasive biopsy will be performed immediately prior to 3 mm punch biopsy. For exploratory analyses, an additional 4 mm punch biopsy may be obtained at the time of diagnostic sampling from all lesions not excised surgically. A small volume of local anaesthetic will be injected prior to tissue sampling to maintain comfort. Allergic reactions from mild to severe may occur in response to any constituent of the local anaesthetic agent (these are rare). The skin will be disinfected prior to any biopsy. The risks of biopsy include pain, swelling, bleeding and infection. In the event that an infection occurs, antibiotics will be prescribed for treatment that do not interact with treatment required for BU. Biopsy sites will have a wound closure strip and bandage applied, healing to form a small scar.

### Allergic reaction

Allergic reactions from mild to severe may occur in response to any constituent of the challenge agent or antibiotic, including any excipient used in manufacture of the challenge agent. Participants with any history of allergy to any of the excipients used for the challenge manufacture will be excluded. An initial ‘sham’ challenge will be administered prior to the infectious challenge, and the challenge will only proceed if no clinically significant allergic reaction is identified. Suitably qualified personnel, with access to emergency first aid equipment, will be present during and for 4 hours after each challenge.

### Risk to participant contacts

There are minimal ‘third party’ risks, as human-to-human transmission is not thought to occur ^93^. Nevertheless, participants with open wounds will receive dressings to cover the wound to minimise environmental contamination. As the study is taking place in Victoria, Australia, where the disease is already endemic, there is no excess risk of introducing the agent into the environment, and there is no evidence in Australia that humans introduce the organism into the environment. A full whole genome sequence will be published in the event that comparison to other *M. ulcerans* sequences is required.

### Risk to researchers

Risks to researchers include the possibility of needle stick injury and accidental splash of the challenge agent during manipulation or injection. Trained staff (nurses and doctors) will perform all procedures that are within their scope of practice. Personal protective equipment will include disposable gowns, gloves and protective eyewear.

### Risk of unexpected participant pregnancy

*M. ulcerans* is not transmitted vertically. Rifampicin is known to reduce the effectiveness of hormone-based contraceptives such as the oral contraceptive pill, necessitating the use of alternative methods for people of child-bearing age ^94^. During the trial and for 30 days after the last dose of any antibiotic, one acceptable method of contraception ( Table 4) will be required. Participants who become pregnant after the challenge will only continue trial procedures required for safety analysis and clinical monitoring. If appropriate, prompt surgical excision will be the suggested treatment option should a lesion develop. In Australia, guidelines recommend the combination of rifampicin and clarithromycin to treat BU in pregnancy ^90^. Maternal use of rifampicin is not associated with an increased risk of congenital malformations or adverse pregnancy outcomes, but is considered a category C medication in pregnancy ^95^. In the final trimester, there is increased risk of haemorrhagic disorders of the newborn, so vitamin K supplementation to the mother is recommended in the last 4 – 8 weeks of pregnancy ^95^. Clarithromycin (category B3) is considered ‘safe to use’ outside of the first trimester by local Australian guidelines ^95^, although meta-analyses of clarithromycin during pregnancy suggest an association with poor pregnancy-related outcomes, including spontaneous abortion ^96^. This underscores the importance of screening all candidate participants of childbearing potential for pregnancy at entry and again prior to antibiotic commencement, in addition to ensuring they are aware of the need for acceptable contraception. In the unlikely event that pregnancy occurs during the study, the trial team will collect pregnancy-related information from all pregnant participants, and the participant/s will be followed up to determine the outcome of the pregnancy.

**Table 4.** Acceptable methods of contraception for people of childbearing potential.

Sexual abstinence and abstinence from heterosexual intercourse (for people with female sexual reproductive organs) (periodic abstinence and withdrawal methods are not acceptable forms of contraception)
Bilateral oophorectomy and/or hysterectomy
Bilateral tubal ligation
Copper intra-uterine device (IUD)
Levonorgestrel-releasing IUD (e.g., Mirena, Skyla)
Male condom and occlusive cap (diaphragm or cervical/vault cap) with spermicidal foam / gel / film / cream / suppository
Vasectomised partner (if sole partner)

Recommendations are based on the Clinical Trials Facilitation and Coordination Group recommendations related to contraception and pregnancy testing in clinical trials ^97^, excluding hormone-based contraceptives options due to potential drug interactions with rifampicin and/or clarithromycin. The contraceptive effects of levonorgesterel-releasing intra-uterine systems are unlikely to be effected by drug interactions, as the direct release of levonorgestrel into the uterine cavity is unlikely to be affected by drug interactions via enzyme induction ^98^.

Recommendations are based on the Clinical Trials Facilitation and Coordination Group recommendations related to contraception and pregnancy testing in clinical trials ^97^, excluding hormone-based contraceptives options due to potential drug interactions with rifampicin and/or clarithromycin. The contraceptive effects of levonorgesterel-releasing intra-uterine systems are unlikely to be effected by drug interactions, as the direct release of levonorgestrel into the uterine cavity is unlikely to be affected by drug interactions via enzyme induction ^98^.

## Safety reporting

### Safety definitions

Safety definitions are aligned with the National Health Medical Research Council (NHMRC) document ‘Safety monitoring and reporting in clinical trials involving therapeutic goods.’ An adverse event (AE) is defined as any untoward medical occurrence in a participant that does not necessarily have a causal relationship with the intervention. An adverse reaction (AR) is defined as any untoward and unintended response related to the challenge agent or treatment.

A serious adverse event (SAE) is defined as any event that results in death, is life-threatening, requires inpatient hospitalisation or prolongation of existing hospitalisation, results in persistent or significant disability or incapacity or is a congenital anomaly or birth defect. A serious adverse reaction (SAR) is a serious adverse event that is attributed to a trial challenge agent or treatment. A suspected unexpected serious adverse reaction (SUSAR) is a serious adverse reaction likely due to a challenge agent or treatment, but is not consistent with known (or expected) information about the challenge agent or treatment. An adverse event of special interest (AESI) is any adverse event that may be related to the challenge agent, with an unexpected event or outcome (whether serious or non-serious). These events may warrant further investigation in order to characterise and understand the event.

### Expected Adverse Events (AEs)

*Expected AEs due to challenge:*

- Pain/tenderness at or near challenge site
- Redness at or near challenge site
- Swelling at or near challenge site
- Scaling at or near the challenge site
- Pustule at or near the challenge site
- Nodule at or near the challenge site
- Ulceration at or near the challenge site
- Scar at or near challenge site

*Expected AEs due to biopsy:*

- Pain/tenderness at biopsy site
- Redness at biopsy site
- Swelling at biopsy site
- Scar at biopsy site

*Expected AEs due to antibiotic therapy:*

- Nausea
- Discoloration of bodily fluids
- Dysgeusia
- Bloating and/or dyspepsia
- Loose stool or frequent bowel motions
- LFT derangement < 5x upper limit of normal
- Prolongation of QTc interval ≤ 20 milliseconds ^99^

### Grading and outcome of Adverse Events

Excluding scarring, all adverse events that are probably and definitely related to challenge or treatment, whether serious or not, which persist at the end of the study will be followed up by the study team until resolution. Additional follow-up visits may be arranged to enable this. Participants with AEs may be advised to consult their GP, or the study team will arrange specialist review at their local public healthcare service. Grading criteria for local reactions are outlined in Table 5, and other adverse events will be graded as per the Common Terminology Criteria for Adverse Events (CTCAE) ^100^.

**Table 5.** Grading of local adverse events (adapted from 102).

Adverse event	Grade	Measurement
Pain/tenderness at injection or biopsy site	0 1 2 3	No pain Tender to touch only Painful on movement Persistent severe pain at rest
Redness at injection or biopsy site	0 1 2 3	0 mm 1–50 mm 51–100 mm > 100 mm
Swelling at injection or biopsy site	0 1 2 3	0 mm 1–20 mm 21–50 mm > 50 mm
Scaling/pustule/nodule at challenge site	0 1 2 3	0 mm 1–15 mm 16–29 mm ≥ 30 mm
Ulceration at challenge site	0 1 2 3	0 mm 1–9 mm 10–19 mm ≥ 20 mm, or ≥ 3 ulcers at the challenge site
Scarring at challenge site	0 1 2 3	0 mm 1–9 mm 10–19 mm ≥ 20 mm

The following information will be documented for all adverse events: description, date of onset and end date, severity and any treatment or intervention undertaken. The severity of AEs will be assessed using the following scale: 1 = mild, 2 = moderate, 3 = severe.

*The outcome of all AEs will be assessed as:*

- Recovered/resolved without scarring
- Recovered/resolved with scarring
- Recovered/resolved with sequelae (non-scar)
- Ongoing at study completion
- Fatal
- Unknown

*Causality assessment:*

A causality assessment will be performed for each AE according to the following definitions:

(0) No relationship: No temporal relationship to intervention and/or definite alternative aetiology for the AE is identified

(1) Unlikely related: Unlikely temporal relationship to intervention, and/or an alternate aetiology is more likely

(2) Possibly related: There may be a temporal relationship to the intervention, and/or an alternate aetiology is less likely

(3) Likely (or probably) related: Reasonable temporal relationship to intervention, and/or event not readily explained by alternative aetiology

(4) Definitely related: Reasonable temporal relationship to intervention and/or event not readily explained by alternative aetiology.

The chief investigator, in consultation with the study steering committee (SSC), will determine causality of AEs. Definite (4), probable (3) and possible (2) are considered to be related. No relationship (0) and unlikely (1) are unrelated.

BU lesions have previously been classified into severity categories according to the WHO ^101^. All participants are anticipated to develop category I lesions (i.e., single lesions < 5 cm in diameter), but because this trial is designed to treat participants relatively promptly after a lesion is reported, more stringent severity grading will be implemented (see Table 5). A cellulitic or oedematous lesion will be defined as erythema and/or induration and/or oedema advancing > 5 cm from the challenge site ≤ 7 days from the lesion being reported.

### Study Management Team

A study management team (SMT) will be responsible for routine day-to-day management decisions during the study, including medically-qualified investigators and research staff. They will be responsible for reviewing participant diaries and assessing participants during scheduled follow-up. They will also be available for any *ad hoc* assessments, with a member of the SMT on-call to respond to any participant concerns during the trial. The SMT will report to the study steering committee (SSC) during scheduled or as-required meetings.

### Study Steering Committee

The SSC will include experts involved in all aspects of trial design and protocol development. Trial officers, managers, scientists and clinicians may be members of the steering committee. The steering committee will have a range of relevant experience, including the diagnosis and treatment of BU, and success in establishing ethical and safe controlled human infection trials. The committee will meet regularly to discuss the trial design, implementation, outcomes and any adverse events. They will also meet to discuss and implement recommendations provided by the data safety review committee.

### Safety Review Committee (SRC)

The SRC is an independent committee which will review safety data for the duration of the trial. All roles and responsibilities of the SRC will be outlined by specific terms of reference. The SRC will be supplied with a safety report at the end of Stage 2A of the study, before dose escalation (if applicable) and dose confirmation, in the event of an SAE/SUSAR/AESI, or if requested at any time by any SRC committee members. The specific role of the SRC is to:

- Independently review SAEs, SUSARs, and AESIs regardless of relatedness to any of the study procedures throughout the study,
- Review whether study objectives are met, including confirming the presence of *M. ulcerans* in clinical lesions at the challenge site
- Perform unscheduled reviews on request of the study team as required, depending on the frequency and severity of reported adverse events
- Provide counsel where the study team feels independent advice or review is required.

### Discontinuation

The study will discontinue enrolment in the event that the MuCHIM has an unacceptable safety profile, as determined by the SSC, in collaboration with the SRC. Participants already challenged may be offered pre-emptive antibiotic therapy if they meet STOP criteria. Participants will continue to receive follow-up according to Study Procedure.

### STOP criteria

For participants who are already enrolled and have been challenged with *M. ulcerans* JKD8049, the STOP criteria may be implemented, with the approval of the SSC in collaboration with the SRC, due to:

- Medical illness: STOP if they cannot continue to be involved in the trial due to a medical condition that arises during the course of follow-up and may result in poorer outcomes if allowed to progress (e.g., malignancy requiring chemotherapy)
- Engagement: STOP if the trial team have concerns about the participant’s ability to commit to safety checks and frequent communication.
- Study is discontinued for any other reason.

Participants who meet the STOP criteria will be offered pre-emptive antibiotic treatment and follow-up will continue as per ‘Study Procedure’, with participants encouraged to participate in all safety interventions (or if not possible, the participant will be linked into care with their GP, with additional phone or email follow-up, if feasible).

### Protocol registration and approval

In Australia, challenge agents are not considered therapeutic products, so although these are not regulated by the Therapeutic Goods Administration (TGA), the TGA’s Clinical Trial Notification (CTN) scheme provides an avenue through which ‘unapproved’ therapeutic goods can be used for experimental purposes in humans. A CTN will therefore be completed for the challenge agent *M. ulcerans* JKD8049, with dose manufacture following the principles of Good Manufacturing Practice. Following peer review of this provisional protocol, and after community/stakeholder engagement, a refined protocol incorporating recommendations will be submitted for review by an Australian Institutional Review Board (Human Research Ethics Committee) in accordance with the National Statement on Ethical Conduct in Human Research. It will then be registered on the Australian New Zealand Clinical Trials Registry ( www.anzctr.org.au). The sponsor is the University of Melbourne, and the study will be indemnified under an existing institutional insurance policy. The results of the study will be of national and international significance. The members of the investigator committee have established links to numerous stakeholder groups, with national and international profiles that will ensure dissemination of the results in peer-reviewed journals and presentation at relevant conferences.

### Publication policy

Any publication related to the trial will be consistent with the Consort Guidelines and checklist ( http://www.consort-statement.org/) and will be based on the International Committee of Medical Journal Editors (ICJME) requirements for authorship. The findings of this trial will be disseminated amongst the scientific community. The findings of this trial will be submitted for peer review and publication in scientific journals and presented at appropriate local, national and international conferences. A lay report of findings will be made available to all participants.

## Discussion

MuCHIM, the pioneering mycobacterial human infection model detailed in this report, has the potential to fast-track development of vaccines and new therapeutics for BU. Providing all reasonable measures are implemented to prioritise participant safety, there is ethical justification to establish such a model. By accelerating our ability to prevent BU, many individuals visiting and living in endemic communities, and those in communities which may yet become endemic, may benefit from this research tool. It is unlikely that other approaches to testing BU vaccines in humans will be successful in any reasonable timeframe.

There are aspects of this model that make it distinct from most previously reported CHIMs. Firstly, it is exclusively an outpatient model, due to the very slow natural history of BU, including its long incubation period and the typically slow onset of disease and subsequent healing. Although this is a logistical challenge, if the known natural history of BU is adequately replicated (e.g., incubation period of 4 – 5 months), this will establish the model’s generalisability. Secondly, the need for a prolonged course of antibiotics to treat the challenge infection necessitates additional safety conditions, including cautious patient selection, close outpatient monitoring for adverse events, and surgical options in case of antibiotic intolerance. A strength of our study design is that we can enrol our target therapeutic population (Australian adults); the majority of people with BU in Australia are adults ^103^, unlike in African settings, where children often present with BU ^104^.

A benefit of CHIMs is the ability to test numerous interventions simultaneously within a single trial. For example, if 20 participants are recruited, 10 may be randomly allocated a vaccine with some reasonable pre-clinical evidence of efficacy. Of those who develop infection, experimental treatment approaches within groups can then be tested, with the option of proven, effective antibiotic treatment in the event that the experimental treatment is unsuccessful. Additional information gathered during these trials, including a clear characterisation of the natural history and immune response to early BU, will contribute valuable scientific data to identify correlates of protection, informing and refining the design of future vaccine targets. In a disease such as BU, where timing of exposure is difficult to define in the field, MuCHIM is a very powerful research platform that will allow us to define correlates of protection.

Risks of this model include the need for prolonged antibiotic duration to treat BU, rendering the trial susceptible to additional risks related to antibiotic side effects. The common antibiotic side effects are well understood and manageable. There are very few extremely serious side effects, and their occurrence is very rare. To mitigate these risks, MuCHIM includes rigorous safety considerations, including selection criteria that reduce the probability of encountering side effects, minimising the duration of antibiotic treatment, and telemedicine to facilitate the follow-up required. Applying a comprehensively characterised challenge isolate, administered as an accurate, very low yet realistic dose, further adds to this favourable safety profile.

As for any CHIM ^16^, MuCHIM may lack generalisability in other contexts, such as to BU in Africa, as there are challenge strain and host differences. Although *M. ulcerans* JKD8049 is classified phylogenomically within the same ancestral lineage as African *M. ulcerans* isolates, microbiological differences may include variation in the proportions of mycolactone congeners, and the host’s response to infection. To ensure variations in host genetics, and related immunological responses to *M. ulcerans* infection are accounted for, we will endeavour to ensure participants represent diverse ethnic origins. To note, *M. ulcerans* infection is capable of causing infection in people of any age and ethnicity, and does not clearly discriminate between people with comorbidities or other risk factors such as immunocompromise. The clinical and immunological data gathered from healthy and young adult volunteers may not necessarily be generalisable to people of all ages. Unpredictable individual variation is a recognised limitation of CHIMs in general, due to the small sample sizes used; this necessitates further investigation of promising interventions in larger clinical trials.

There are some limitations which balance the generalisability of the model with the focus on optimising participant safety. Early diagnosis and prompt treatment, although designed to minimise risk to participants, will only allow the model to characterise the earlier stages of the disease and related immunological responses to infection. Although some lesions are known to spontaneously resolve in the field, all visible lesions will be treated with antibiotics in this model, and therefore some correlates of protection may remain unrecognised. Finally, immunological responses to infection may also be altered in response to excisional or punch biopsy, particularly in early lesions.

Preventing disease in humans is just one arm of the broader ‘One Health’ approach ultimately required to stop Buruli ulcer; collateral and synergistic strategies will need to reduce the burden of disease in native possums ^105^ and control the *Aedes notoscriptus* mosquito vector ^11^. There may be equipoise for a human mosquito blood-feeding CHIM arm in future applications of this research, which may conclusively demonstrate the successful transmission of *M. ulcerans* to humans by mosquito bite. This challenge route also has the advantage of accounting for unrecognised mosquito-related pathogenicity and/or transmission factors. A mosquito route of infection will also ensure a biologically relevant dose of *M. ulcerans* is received by participants, but this may be difficult to control. Vector transmission has been incorporated into CHIMs previously, including in controlled malaria infection using mosquitos ^106^, and recently with a protocol to introduce *Leishmania major* into humans via phlebotomine sand fly bite ^107^. Nevertheless, MuCHIM attempts to closely imitate the most likely transmission condition in Australia, and if successful, a mosquito blood-feeding arm may not be required; this would circumvent the risks related to mosquito-bite allergy and enable a more practical challenge protocol.

If successful, this trial will finally overcome numerous scientific hurdles to understanding this disease, including the reliance on retrospective observational methodology to understand complex human immune responses to *M. ulcerans* infection ^15^ and the limitations of animal models and the heterogeneity in approaches to test vaccine efficacy ^6^. Once established, MuCHIM could deliver the first evidence of human BU vaccine efficacy since *M. bovis* BCG was first trialled in the 1960’s ^8^, underscoring why ambitious and novel advances are required to prevent 50 years of further inertia in BU vaccine development.

## Ethics and consent

Ethical approval and consent were not required for this provisional protocol.

## Data Availability

No data are associated with this article. Figshare: SPIRIT checklist for “A human model of Buruli ulcer: Protocol for an initial *Mycobacterium ulcerans* controlled human infection study”. https://doi.org/10.6084/m9.figshare.26361901.v1 ^108^ Data are available under the terms of CCBY4.0 licence.
