# Supplementary material for: A human model of Buruli ulcer: Provisional protocol for a Mycobacterium ulcerans controlled human infection study
Source: Wellcome Open Res. 2024 Oct 21;9:488. Originally published 2024 Aug 19. [Version 2] doi: 10.12688/wellcomeopenres.22719.2 (PMC11462124; doi:10.12688/wellcomeopenres.22719.2)
Supplement: Peer review history

# Reviewer report: Reviewer response for version 2

**DOI:** 10.21956/wellcomeopenres.25647.r106850

**Published:** 2024-11-14

**Authors:** Louisa Warryn

**Affiliations:** 1 Swiss Tropical and Public Health Institute, Allschwil, Switzerland, Allschwil, Switzerland

The authors have addressed most of my previous comments. There is only the statement regarding the *M. ulcerans *lineage: " *Although M. ulcerans* *JKD8049 is classified phylogenomically within the same* *ancestral lineage as African M. ulcerans isolates, microbiological differences may include variation in the proportions of mycolactone congeners, and the host’s response to infection*."

 To avoid any confusion, I would recommend the authors reword this statement, because African and Australian *M. ulcerans *isolates belong to the classical lineage, while the ancestral lineage includes isolates from Japan, for e.g.

Is the study design appropriate for the research question?

Partly

Is the rationale for, and objectives of, the study clearly described?

Yes

Are sufficient details of the methods provided to allow replication by others?

Partly

Are the datasets clearly presented in a useable and accessible format?

Not applicable

Reviewer Expertise:

NA

I confirm that I have read this submission and believe that I have an appropriate level of expertise to confirm that it is of an acceptable scientific standard.

# Reviewer report: Reviewer response for version 2

**DOI:** 10.21956/wellcomeopenres.25647.r108497

**Published:** 2024-11-06

**Authors:** Aloysius Loglo

**Affiliations:** 1 Kumasi Centre for Collaborative Research in Tropical Medicine, Kumasi, Ghana, Ghana

The authors have created a well-thought-out and detailed protocol for a controlled human model of *Mycobacterium ulcerans* (MU) infection. Overall, this study is well-designed and clearly explained. Every possible risk with a well-explained mitigation step has been outlined. Even though this CHIM has not been tested in MU research, it is clear that the study has the potential to impact our understanding of the disease significantly. However, a minor aspect of the protocol needs reconsideration or further input to ensure clarity of information.

 First, the amount of blood the authors intend to obtain from each participant needs to be reconsidered, as 450mL in three months is a lot.

 Second, in the exploratory analyses section (page 19), the authors have not clearly stated the inflammatory cytokines and chemokines that will be measured for the ELISA and PBMCs. There are a lot of well-studied Buruli ulcer markers from the literature that could be considered when making this decision.

 Third, in the “understanding the host microbiome over time “section, could the authors explain a bit more about how the skin swab and faecal swab samples will be obtained?

 Finally, in the discussion section, please correct the spelling of “mosquitos”

Is the study design appropriate for the research question?

Yes

Is the rationale for, and objectives of, the study clearly described?

Yes

Are sufficient details of the methods provided to allow replication by others?

Yes

Are the datasets clearly presented in a useable and accessible format?

Yes

Reviewer Expertise:

Clinical Immunology of Mycobacterium ulcerans

I confirm that I have read this submission and believe that I have an appropriate level of expertise to confirm that it is of an acceptable scientific standard.

# Reviewer report: Reviewer response for version 2

**DOI:** 10.21956/wellcomeopenres.25647.r106852

**Published:** 2024-11-01

**Authors:** Paul J Converse

**Affiliations:** 1 Department of Medicine, Johns Hopkins University Center for Tuberculosis Research, Maryland, USA

The author addressed all my comments. Approved

Is the study design appropriate for the research question?

Yes

Is the rationale for, and objectives of, the study clearly described?

Yes

Are sufficient details of the methods provided to allow replication by others?

Yes

Are the datasets clearly presented in a useable and accessible format?

Yes

Reviewer Expertise:

Mouse model of M. ulcerans infection and treatment optimization

I confirm that I have read this submission and believe that I have an appropriate level of expertise to confirm that it is of an acceptable scientific standard.

# Reviewer report: Reviewer response for version 1

**DOI:** 10.21956/wellcomeopenres.25021.r97141

**Published:** 2024-10-08

**Authors:** Yaw Ampem Amoako

**Affiliations:** 1 Kumasi Centre for Collaborative Research, Kumasi, Ghana

The authors present a protocol for studying BU using a controlled human infection model. This is a bold attempt with enormous potential applications if it succeeds. The study is well designed and described. The potential risks and mitigation strategies are well outlined. A potential concern is the volume of blood to be collected during the study period. As indicated however, this may pose only minimal risks to healthy volunteers. If suitable volunteers are identified, the authors have the requisite expertise to actualize the protocol and manage the study.

Is the study design appropriate for the research question?

Yes

Is the rationale for, and objectives of, the study clearly described?

Yes

Are sufficient details of the methods provided to allow replication by others?

Yes

Are the datasets clearly presented in a useable and accessible format?

Yes

Reviewer Expertise:

Drug treatment and clinical aspects of Buruli ulcer (BU) disease

I confirm that I have read this submission and believe that I have an appropriate level of expertise to confirm that it is of an acceptable scientific standard.

# Response

**Published:** 2024-10-09

**Authors:** Stephen Muhi

We thank Dr Amoako for their support.

# Reviewer report: Reviewer response for version 1

**DOI:** 10.21956/wellcomeopenres.25021.r97138

**Published:** 2024-10-02

**Authors:** Paul J Converse

**Affiliations:** 1 Department of Medicine, Johns Hopkins University Center for Tuberculosis Research, Maryland, USA

The authors have presented a detailed and cautious protocol for studying Buruli ulcer (BU) in a controlled human infection model. They have identified many potential issues that could arise in the selection of human volunteers to assure that the study can be as low risk as possible. Given the apparently high awareness of BU in Victoria State, Australia, they may indeed succeed in finding volunteers to be infected with the well-characterized, drug-susceptible, *M. ulcerans *strain they have described in previous publications. The volunteers, however, must also not reside too near an already endemic area.

 One weakness in the proposal is the plan to study immunological responses or conduct immunological analyses. These do not appear to be functional studies but rather the assessment of cellular (and cytokine) phenotypes which may not provide a great deal of information. The authors should propose functional studies that may bear fruit. They should also review the studies by Phillips and colleagues in Ghana that attempted to do functional analyses.

Is the study design appropriate for the research question?

Yes

Is the rationale for, and objectives of, the study clearly described?

Yes

Are sufficient details of the methods provided to allow replication by others?

Yes

Are the datasets clearly presented in a useable and accessible format?

Yes

Reviewer Expertise:

Mouse model of M. ulcerans infection and treatment optimization

I confirm that I have read this submission and believe that I have an appropriate level of expertise to confirm that it is of an acceptable scientific standard, however I have significant reservations, as outlined above.

## References

[1] : Analysis of Mycobacterium ulcerans-specific T-cell cytokines for diagnosis of Buruli ulcer disease and as potential indicator for disease progression. *PLoS Negl Trop Dis* .2017;11(2) : 10.1371/journal.pntd.0005415 e0005415 10.1371/journal.pntd.0005415 28241036 PMC5344519

[2] : Dynamics of the cytokine response to Mycobacterium ulcerans during antibiotic treatment for M. ulcerans disease (Buruli ulcer) in humans. *Clin Vaccine Immunol* .2009;16(1) : 10.1128/CVI.00235-08 61-5 10.1128/CVI.00235-08 19005025 PMC2620668

[3] : Cytokine response to antigen stimulation of whole blood from patients with Mycobacterium ulcerans disease compared to that from patients with tuberculosis. *Clin Vaccine Immunol* .2006;13(2) : 10.1128/CVI.13.2.253-257.2006 253-7 10.1128/CVI.13.2.253-257.2006 16467334 PMC1391935

[4] : Immunosuppressive signature of cutaneous Mycobacterium ulcerans infection in the peripheral blood of patients with buruli ulcer disease. *J Infect Dis* .2009;200(11) : 10.1086/646615 1675-84 10.1086/646615 19863437

# Response

**Published:** 2024-10-09

**Authors:** Stephen Muhi

We thank Dr Converse for the thoughtful suggesion. We agree that functional assessment of immune function is important to consider, and have updated the manuscript accordingly.

# Reviewer report: Reviewer response for version 1

**DOI:** 10.21956/wellcomeopenres.25021.r97136

**Published:** 2024-09-25

**Authors:** Louisa Warryn

**Affiliations:** 1 Swiss Tropical and Public Health Institute, Allschwil, Switzerland, Allschwil, Switzerland

The authors have provided a very considered protocol for a CHIM for *M. ulcerans *infection. They have taken steps to address the peculiarities of *M. ulcerans *infection, including the slow progression and lengthy antibiotic treatment, that make a MuCHIM different from other reported CHIM. They have additionally outlined the limitations of such a model. There are however some points that the authors should provide additional clarification on.

  

1. While the authors give a clear enough reason why they intend to develop a CHIM, in my opinion they may be underestimating the usefulness of animal models for BU vaccine development, especially since animal studies have led to many of the advances that aid in BU control today. Mouse studies have been instrumental in developing the recommended antibiotic regimen, and most of the insights we have today regarding the pathogenesis and molecular targets of *M. ulcerans* were gotten using animal models. It does seem to me that it is entirely possible to do BU vaccine studies in suitable rodent models as well. I feel it is somewhat unfair to aggregate data from disparate vaccine studies done in mice and use that as an argument against all such studies. In my opinion, that rather calls more for standardised animal studies using well-defined parameters than it does for developing a CHIM. That said, I agree with the authors that a CHIM in and of itself “could be an efficient platform for evaluating vaccines, chemoprophylaxis, and novel therapeutics”.
  2. In the section regarding “Study design”:

- Scarring from successful resolution of infection seems to be a given, rather than a possibility as the authors seem to suggest (perhaps I read that wrong), whether it is from the lesion itself, from biopsies, or from excision treatment. I think this point should be clearly stressed to prospective participants.
- Please clarify what “Temporary exclusion criteria” mean. Would such temporarily excluded individuals be allowed to participate afterwards?
- On page 6, it is stated that “Written informed consent will be obtained by the responsible clinician on the day of any other procedure, including punch biopsy and therapeutic excisional biopsy.” However, Table 3 seems to indicate all additional consent will only be verbal. Please harmonise this.
- Please consider also including these points in the eligibility criteria if applicable: (i) presence of tattoos on the proposed inoculation sites, (ii) previous exposure to *M. marinum*, (iii) tolerance to analgesics given the potentially painful study procedures (only tolerance to local anaesthetics is mentioned).
- As a general question, how exactly will previous BCG vaccination be determined? Would it be simply by looking for the vaccine scar, or would previous vaccination records be necessary? I only ask because I think some of your target population may have similar scars from smallpox (or Mpox) vaccination.
- Please harmonise the full meaning of LSA. It is variously given as “laboratory safety analyses” and “laboratory safety assessments”.
- In Table 2, it would be better to merge both “Parameter” columns.
- In the subsection “Challenge strain manufacture and cell banking”, it may also be helpful, at least in the initial trials, to determine CFU of the final inocula to ascertain that the calculated CFUs were indeed what were inoculated.

         3.Please address these points in Table 3.

- It is unclear to me what the “Time window” indicates. Please clarify this.
- In Table 3A, if boxes shaded in light red are the in-person visits, how can you sample for LSAs during visits that are not in-person? Perhaps you have omitted to colour some boxes accordingly?
- Please mention in the main text when verbal consent is done (there is currently no mention of verbal consents in the main text).
- In Table 3B, please correct spelling of “fortnightly” and “virtual”.
- In Table 3C, please correct spelling of “faecal”.
  4. In the subsection “Case definition”, you mention testing samples from the challenge site (swab or biopsy) for the presence of IS *2404* via PCR as the current diagnostic gold standard. Would you consider also performing additional tests, since the DNA from the challenge might linger and give positive results? Perhaps looking for known histological hallmarks of the infection in biopsies (if biopsies are taken), or microbiological culture of samples?
  5. It would be helpful to clearly state what the progression of events will be in the main text, and not only include this in the figures/tables. As it stands, it is not yet clear how soon after lesion development diagnostic sampling will be done and treatment initiated. As these are crucial aspects of the protocol, it is best if the authors clearly state these points in the main text to prevent any ambiguity.
  6. In the subsection “Lesion sampling”, could you clarify why a biopsy (even if minimally invasive) is preferred rather than fine-needle aspirates?  FNAs are established sample types for BU diagnosis while the minimally invasive biopsy is still exploratory in this context. Therefore, a biopsy may be an optional procedure. If a biopsy will be mandatory for all participants, please clearly state that this will be communicated to the participants during informed consent. As it reads now, it appears that a participant who elects to have only antibiotic treatment can refuse to give a biopsy.
  7.Given the possibility, however remote, of tinnitus developing during macrolide treatment, perhaps consider informing participants beforehand. Perhaps it might be necessary to exclude participants with tinnitus, even if they have no hearing impairment, in case the treatment worsens the condition.
  8.On page 25, the Australian and African *M. ulcerans *strains are in the classical lineage not the ancestral lineage. Please correct this.

Is the study design appropriate for the research question?

Partly

Is the rationale for, and objectives of, the study clearly described?

Yes

Are sufficient details of the methods provided to allow replication by others?

Partly

Are the datasets clearly presented in a useable and accessible format?

Not applicable

Reviewer Expertise:

NA

I confirm that I have read this submission and believe that I have an appropriate level of expertise to confirm that it is of an acceptable scientific standard, however I have significant reservations, as outlined above.

# Response

**Published:** 2024-10-09

**Authors:** Stephen Muhi

Thank you for your considered and thoughtful suggestions. Please find our responses below.

 1. We agree that murine research can indeed offer valuable insights with the potential for translational impact. Our group have also tested this challenge strain in a mouse model ( https://doi.org/10.1128/spectrum.00555-24), and we are preparing a separate manuscript describing the innate and adaptive immunological responses to infection in BALB/c and C57BL/6 mice following challenge with JKD8049. We agree with the reviewer that valuable insights can be obtained from mouse infection models; we have therefore amended the text of the manuscript to acknowledge the impact this research has provided.

 2. We agree that this should be highlighted to participants, and have amended the manuscript to reinforce this (line 337). We have also now clarified what ‘temporary exclusion criteria’ mean in the text. We have updated Table 3B, which now has an added line for written consent to be obtained for procedures (i.e., diagnostic sampling) and verbal consent obtained prior to physical examination. This has been harmonised in the text. We agree that tattoos at the challenge site and previous *M. marinum* infection are reasonable exclusion criteria, and have included these in the manuscript. We do not anticipate participants will require more than very short durations of simple analgesia for any procedures (e.g., over-the-counter paracetamol or ibuprofen). Regarding BCG vaccination, participants will be asked if they have ever received a tuberculosis vaccine, with a review of any available vaccination records, and examined for the presence of a scar in the deltoid region. The smallpox vaccine program ceased in Australia in 1980, and the BCG vaccine campaign ceased in 1984-1985. Therefore, at least for Australians who participated in the childhood vaccinated schedule, no individual aged ≤ 45 is likely to have a smallpox vaccine scar, and those ≤ 41 years of age will be unlikely to have a BCG vaccine scar. Regarding 'LSA', thank you for highlighting this inconsistency, we have changed all instances to ‘laboratory safety assessment'. Regarding Table 2: Thank you for the suggestion. In our original manuscript, these columns are indeed merged into a single column. It appears that the journal’s formatting/typesetting has separated into these columns. We will contact the editorial team to suggest these changes. Regarding CFU count: We do plan to perform this final CFU count to confirm the inoculated dose; we have added this into the manuscript for additional clarity. Nevertheless, our cell bank will undergo quality control measures to ensure the inoculated dose is within the anticipated range.

 3. *Time window* is the acceptable variation in the numbers of days allowed to deviate from the schedule (e.g., we ask participants to complete their study diary on study day 14, although acceptable deviation is study day 12, 13, 15 and 16). We have clarified this in the text Thank you for highlighting the error in Table 3A. We have updated the table accordingly. There should just be 1 in-person study visit monthly until the lesion is reported. Regarding verbal consent: Thank you for identifying this omission. Verbal consent will be required prior to any physical examination. We have updated the manuscript to clarify this. Regarding Table 3B and 3C: Thank you for noting these errors. They were not in the original submitted manuscript, and appear to have been introduced during typesetting. I will inform the journal’s editorial staff (we also noticed the incorrect spelling of ‘weekly’ in Table 3B, which we will also correct).

 4. We indeed do plan to perform these additional tests (culture and histology), assuming there is suitable sample provided (described in ‘Microbiological features of infection’, and ‘Tissue immune responses’).

 5. We have amended the section ‘Expected outcome’ to provide greater clarity: *“If a participant reports a lesion, they will be reviewed by the trial team within 48 – 72 hours. The expected outcome is that an ‘early lesion’ (patch of erythema and/or induration) will develop into a ‘pre-ulcerative lesion’ (nodule/plaque/pustule); in the event that they develop an ‘early lesion’ or ‘pre-ulcerative lesion’, the participant will be asked to monitor the lesion and return for review if ulceration occurs. Diagnostic sampling will be performed, and treatment will be initiated at the onset of any ulceration. If any ‘early lesion’ fails to progress into a pre-ulcerative lesion despite 10 days of monitoring, sampling will be performed, and treatment will be initiated. If any ‘pre-ulcerative’ lesion persists after 7 days without progressing to ulceration, sampling will be performed, and treatment will be initiated to minimise subcutaneous (subclinical) advancement of infection. In summary, the maximum duration of any lesion is 17 days, assuming an ‘early lesion’ progresses into a ‘pre-ulcerative’ lesion on day 10, and does not progress to ulceration *(see ‘Study procedure’ for further detail, and the procedures for other outcomes that are less likely to occur). *Follow-up frequency will increase to weekly for 4 weeks after any lesion is reported.”*

 6. Thank you for your interesting question/comment. FNA is not currently used for suspected BU lesions in Australia, and punch biopsy is used if swabs are negative or for non-ulcerative lesions ( https://www1.racgp.org.au/ajgp/2024/september/an-overview-of-buruli-ulcer-in-australia/). This is likely partially because clinicians do not have experience with the FNA technique, whilst punch biopsy is widely practiced, so practitioners are very familiar with its use. Punch biopsy also gives histopathology to evaluate for an alternative diagnosis, such as skin cancer (which is a far more common problem in the Australian context). In the proposed CHIM, all non-ulcerated lesions will require a small diagnostic punch biopsy (3 mm in diameter), with the minimally invasive biopsy device performed first. The text has been updated to clarify that a diagnostic punch biopsy will be required if the participant develops a non-ulcerative lesion within the stated timeframes.

 7. We agree that tinnitus is a reasonable exclusion criterion and we have amended the text to reflect this point.

 8. Regarding 'lineage', we have amended the text as suggested.
